# Development and Application of the Scale-Up Reflection Guide (SRG)

**DOI:** 10.3390/ijerph20116014

**Published:** 2023-05-31

**Authors:** Karen Lee, Melanie Crane, Anne Grunseit, Blythe O’Hara, Andrew Milat, Luke Wolfenden, Adrian Bauman, Femke van Nassau

**Affiliations:** 1 Sydney School of Public Health, The University of Sydney, Sydney, NSW 2050, Australia; 2 The Australian Prevention Partnership Centre, Level 3, 30C Wentworth Street, Sydney, NSW 2037, Australia; 3 School of Public Health, The University of Technology Sydney, 15 Broadway, Sydney, NSW 2007, Australia; 4 Centre for Epidemiology and Evidence, New South Wales Ministry of Health, 1 Reserve Road, St. Leonards, NSW 2065, Australia; 5 School of Medicine and Public Health, The University of Newcastle, Newcastle, NSW 2308, Australia; 6 Department of Public and Occupational Health, Amsterdam Public Health Research Institute, Amsterdam University Medical Center, Vrije Universiteit Amsterdam, De Boelelaan 1117, 1081 HV Amsterdam, The Netherlands

**Keywords:** scale-up, guide, scaling up, health promotion, chronic disease prevention

## Abstract

Scaling up effective interventions in public health is complex and comprehensive, and published accounts of the scale-up process are scarce. Key aspects of the scale-up experience need to be more comprehensively captured. This study describes the development of a guide for reflecting on and documenting the scale-up of public health interventions, to increase the depth of practice-based information of scaling up. Reviews of relevant scale-up frameworks along with expert input informed the development of the guide. We evaluated its acceptability with potential end-users and applied it to two real-world case studies. The Scale-up Reflection Guide (SRG) provides a structure and process for reflecting on and documenting key aspects of the scale-up process of public health interventions. The SRG is comprised of eight sections: context of completion; intervention delivery, history/background; intervention components; costs/funding strategies and partnership arrangements; the scale-up setting and delivery; scale-up process; and evidence of effectiveness and long-term outcomes. Utilization of the SRG may improve the consistency and reporting for the scale-up of public health interventions and facilitate knowledge sharing. The SRG can be used by a variety of stakeholders including researchers, policymakers or practitioners to more comprehensively reflect on and document scale-up experiences and inform future practice.

## 1. Introduction

Effective health interventions should be scaled up to achieve population-wide benefits [1,2]. The process of ‘scale-up’ or ‘scaling up’ is commonly referred to as “deliberate efforts to increase the impact of successfully tested health interventions so as to benefit more people and to foster policy and program development on a lasting basis” [2]. In the area of health promotion and chronic disease prevention, many interventions are demonstrated to be effective, but are infrequently scaled up or disseminated for wider use [3,4,5]. While various frameworks provide guidance on the optimal steps for scaling up [2,6,7], real-world examples demonstrate that the scale-up occurs through a variety of pathways commonly influenced by the social-political implementation context, resources available for at-scale delivery, and key actors to guide the scale-up process [8,9,10,11,12].

The scientific literature on scale-up consists predominantly of frameworks on ‘how to’ scale up interventions [6,7,13,14], implementation strategies [15,16] and tools for deciding whether an intervention is appropriate or ready for scaling up [17,18]. Many papers on scale-up also describe facilitators and barriers to scale-up [9,19,20], but information on funding, partnership arrangements, delivery mechanisms and decision-making processes from real-world experiences of scale-up is usually absent or inconsistently reported [21]. This limits learning opportunities to inform future scale-up efforts despite calls to improve this reporting process [4,9,22,23,24]. While some descriptions of scale-up may be found in the grey literature, such as government databases or reports, they often lack standardized information to inform replicability [12,25,26,27]. In the peer-reviewed literature in the area of health promotion and chronic disease prevention, such as physical activity or nutrition interventions specifically, publications of scale-up efforts tend to focus on the development and design of the intervention, its characteristics, and results of interventions rather than the processes and context of disseminating and scaling up the intervention [28].

Formal guidance already exists for reporting on a variety of study types, including randomized controlled trials (CONSORT) [29,30], observational or qualitative studies [31,32,33], which facilitates improvements in reporting to enable better appraisal of the quality of the studies while assisting readers to determine applicability to their own context. A similar process for documenting case studies of scale-up has been identified as a gap in the current scale-up literature [25,34,35,36]. Case studies support effective scale-up of health interventions, as they provide an opportunity for rich description of the process of scaling up across various contexts. For this reason, the development of a ‘guide’ for reflecting on and documenting scale-up would be a valuable contribution and may facilitate the development of such case studies [35,37,38].

The purpose of this study was to develop a ‘guide’ for reflecting on and documenting the scale-up of health promotion and chronic disease prevention interventions to aid practitioners, policymakers and researchers undertaking future projects. The new ‘Scale-up Reflection Guide’ includes all important aspects of scale-up that will facilitate more detailed reporting of scale-up experiences to better capture lessons learned. This is critical for building a repository of knowledge to aid replication, understanding, synthesis and learning from previous attempts of scale-up for improving scale-up practices in the future.

In this study, we used health promotion and chronic disease prevention interventions as exemplars to demonstrate the utility of the ‘guide’.

## 2. Materials and Methods

**Figure 1:**
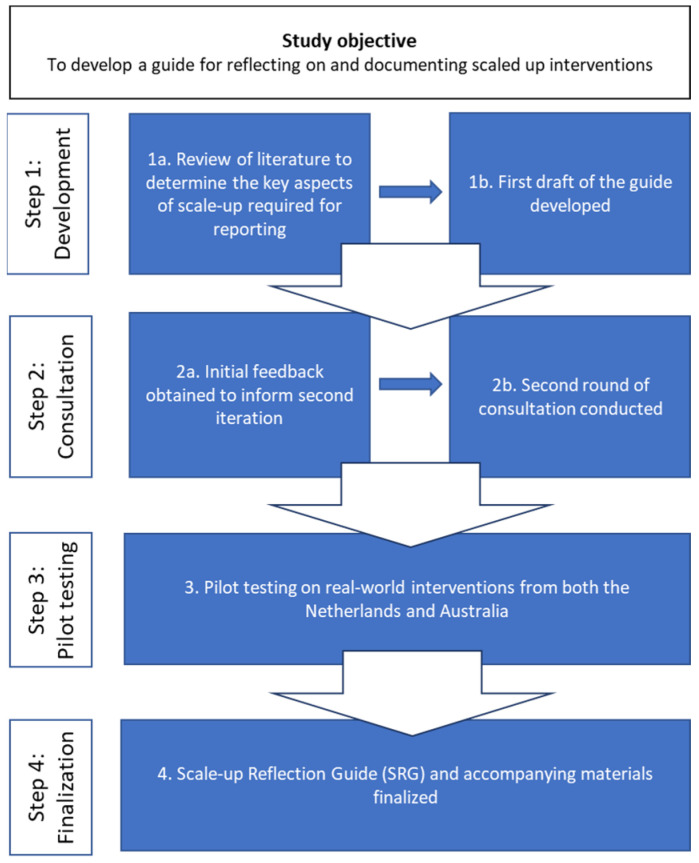
Key steps to developing the Scale-up Reflection Guide.

The development of the Scale-up Reflection Guide (SRG) was an iterative process and comprised (1) reviewing literature, (2) consulting with experts and (3) pilot-testing the guide prior to (4) finalisation. The process is illustrated in Figure 1.

Step 1: Development

We conducted a narrative review to assess current guidance in the literature for developing guidance or case studies in scale-up and, in absence of that, to identify broad scale-up frameworks to identify the key components required for documenting scale-up experiences. The narrative review was conducted over two phases. Firstly, a keyword search of the literature published in English between January 2000–December 2019 was conducted in the OVID MEDLINE database. Two separate search strings were performed separately and subsequently combined. The first search string included the key search terms ‘Case study’ AND [‘Framework’ OR ‘Guide’ OR ‘Checklist’ OR ‘Process model’ OR ‘Tool’] AND [‘Scale-up’ OR ‘Scaling up’]. The second search string included the terms [‘Scale-up’ OR ‘Scaling up’] AND [‘Case study’ OR ‘Framework’ OR ‘Guide’ OR ‘Checklist’ OR ‘Process model’ OR ‘Tool’].

The narrative review was designed by K.L. and M.C., and the abstracts were retrieved and assessed for relevance by K.L. Abstracts were included for full paper review if they met the following inclusion criteria: being published in peer-reviewed literature in English between 2000–2019; providing guidance for developing case studies in scale-up; and describing frameworks, process models, guides or tools associated with scaling-up interventions.

Full papers were retrieved, and relevant papers were assessed independently by two of the authors, K.L. and M.C. Frameworks, guides, tools, process models or checklists were included for further inclusion only if they specified steps or stages to guide the process of scaling up interventions into practice [39] and were applicable to a broad range of health promotion and chronic disease prevention interventions.

Papers on developing case studies were included if they provided specific instruction on the content or process required for collecting and reporting on experiences of scale-up.

Papers were excluded if they described: scale-up and/or evaluation of specific interventions without guidance on how to report scale-up experiences; facilitators and/or barriers to scale-up within a specific intervention or general experiences of scale-up without any framework for guidance; general conceptual issues relating to scale-up; study protocols for potential or existing scale-up; scale-up of biomedical or pharmacological procedures or services and/or medical information technology systems; assessments of readiness for scale-up or scalability; or implementation studies (describing implementation trials and not scale-up).

In addition to the review of the scientific literature, an open keyword search in the Google search engine was conducted, and the results of this search were scanned for relevant articles in accordance with guidance on grey literature searches [40].

Step 2: Consultation

Following the identification of relevant literature, the key concepts from each of the eligible frameworks were extracted and collated by K.L., M.C. and F.v.N. An initial draft of the guide was generated and revised and refined in discussion with the other co-authors as academic experts in implementation and scale-up.

Next, a purposive sample of external experts with academic and policy/practice-based experience of scaling up health interventions (*n* = 4) specifically in the area of health promotion and chronic disease prevention were identified through professional networks in Australia and the Netherlands for further consultation to identify any gaps or areas for improvement as well as to ascertain potential usefulness for users. The consultations in Australia were conducted by K.L. and M.C., and in the Netherlands by F.v.N. A brief topic guide with question prompts was developed to guide the consultations but was employed flexibly to allow for the consultation to be responsive to the respondents. Responses to the question prompts were audio-recorded, and written notes were also taken. Following the consultations, the audio recordings and notes were reviewed and discussed by authors K.L. and F.v.N, and proposed amendments, as suggested through the consultations, were raised with all co-authors, and any changes to the SRG were reached in consultation with and agreed to by all.

Step 3: Pilot testing

Following consultations, the revised SRG was piloted on two real-world examples of scaled-up interventions (Step 3, Figure 1), one from Australia (Get Healthy) and one from the Netherlands (Dutch Obesity Intervention in Teenagers (DOiT)). We selected these interventions, as they target NCD interventions and settings with diverse scale-up processes. The pilot also examined the practical issues in completing the SRG.

Step 4: Finalisation

Following the pilot testing, minor amendments were made based on the pilot experience, and all the SRG and its accompanying materials were finalised.

## 3. Results

Step 1a: Results of narrative and grey literature review

The initial search in OVID MEDLINE with the search terms and search term combinations yielded a total of *n* = 1246 abstracts and an additional *n* = 5 through grey literature. Following the removal of duplicates (*n* = 59), there were a total of *n* = 1192 abstracts to be reviewed as part of Phase 1 (See Supplementary File S1).

Following assessment against review criteria, a further *n* = 1149 were excluded. Forty-three full papers and reports were reviewed against the inclusion criteria for Phase 2. From this, 26 papers were excluded, as eight of them described facilitators of and barriers to scale-up, while a further six described the scale-up of specific interventions, and the remainder either discussed conceptual components of scale-up (*n* = 4), provided assessments of readiness for scale-up (*n* = 3), described the implementation of an intervention (*n* = 1) or pathways for scale-up (*n* = 1), provided a framework to plan for scale-up (*n* = 1) or articulated a knowledge translation model, within which scale-up was flagged as a step (*n* = 1). One article provided guidance for reporting on interventions for publication but was not specific to scale-up and was therefore excluded.

Step 1b: Development of the initial Scale-up Reflection Guide (SRG)

From the review of peer-reviewed and grey literature (*n* = 17), we identified scale-up process frameworks (*n* = 14) and one report providing guidance on how to document scale-up case studies [12]. The remaining *n* = 2 papers [19,41] presented results of their own reviews of scale-up frameworks and provided an additional four frameworks not identified in this literature search.

Upon detailed review of each of these 18 scale-up frameworks and one case study guidance, seven were applicable to a broad range of public health interventions and were deemed useful for developing the key components of the SRG (see Supplementary File S2). Of the remaining frameworks, three were frameworks for determining the scalability or implementation of an intervention rather than providing a process for scale-up, and one was a review of scale-up case studies in physical activity. The remaining eight frameworks were designed for the scale-up of interventions targeting specific conditions, such as HIV or maternal and child health or health technologies.

Other frameworks that focused on implementation outcomes [15,16], sustainability [42,43] or adaptation [44,45,46] were not examined in detail but contributed to supporting information about elements of the guide (see Supplementary File S3).

The one paper that provided guidance on case studies for scale-up did not present a structure or framework for the development of the case study; rather, it posed a series of 20 questions that the author believed any scale-up case study needs to answer [12]. These questions were taken into consideration along with the remaining six frameworks that proposed similar sequential steps for scale-up [2,6,7,11,14,47]. There was considerable agreement on the important aspects influencing scale-up, such as intervention, context, decisions for scaling up, service delivery, scale-up workforce, scale-up and implementation strategy, monitoring and evaluation, facilitators and barriers and sustainability (Table 1). Four frameworks included all nine aspects [2,6,7,14], and the remaining two included seven [11] or eight [47] (Table 1). The twenty questions posed by the scale-up case study guide encompassed all the aspects identified in Table 1.

Key explanations underpinning these nine common aspects of scale-up across the frameworks were also identified (Table 2). These provide clarification of the different aspects of scale-up and identified additional concepts or activities that relate to those aspects. These nine aspects were used to guide the basis of the development of the final SRG (Table 3).

Step 2: Consultations

Of the four advisors consulted, three classified themselves as fulfilling dual roles as policymakers and academics, and one was involved directly in scale-up as a policymaker at the community level in the area of health promotion and chronic disease prevention. The consultations with scale-up experts indicated that, first, there needed to be greater emphasis on documenting adaptations and/or modifications to the intervention and processes for scale-up over time [7]. Second, the different costs associated with scale-up as well as any financial resources made available for scale-up and delivery should be reported separately. Finally, it was recommended that the specific target population for the intervention be clearly described, along with descriptions of the implementation process and scale-up process.

Step 3: Pilot Testing

Following revision, the SRG was tested on two real-world health promotion and chronic disease prevention interventions that had already been scaled up in the Netherlands and Australia independently by authors K.L. and F.v.N. As the aim of this pilot was to test the utility of the SRG, both authors collated the information for each of these real-world interventions based on a range of publicly available and internal documents on the interventions. The ease of completion of the SRG relating to its sense, format and structure, given the available information on each intervention, was particularly examined. As a result of this testing, minor amendments were made to the format and structure of the SRG to improve the user experience (Step 4, Figure 1). Both authors noted that the information required to complete all sections of the SRG was readily available through both public sources and internal documents for the interventions. The content generated for each intervention is provided in Supplementary File S4.

Step 4: Finalization
The Scale-Up Reflection Guide (SRG)

In this section, we provide a structure of how a reflection and documentation of a scale-up experience could be organised (Table 3), followed by suggested steps on how to complete it. The table below provides the detailed sections contained in the “SRG” along with a description of the information required for each of the elements in the section. The rationale for the inclusion of each of the key sections and related elements can be found in Supplementary File S3.

Suggested activities to complete the SRG

The SRG has been designed for a range of users such as researchers, policymakers or practitioners who may have different purposes for completing the SRG. For some, they may want to learn about the scale-up of similar interventions and would complete the SRG by researching a range of information sources on the intervention. Those directly involved in the scale-up process of an intervention may use the SRG to reflect on and document their own experiences for the purposes of developing a case study for publication or reporting. Given the potential diversity in users and/or purposes, we have outlined below some proposed activities for completing the SRG. We recognise that the process is not linear and that the activities are not mutually exclusive. Depending on who is completing the SRG, it is likely that there may be varying levels of familiarity with the intervention which may influence the completeness of the SRG. We have included some suggestions for additional data collection to assist those completing the SRG if they have not been closely involved in the actual scale-up, or where there is limited information about the program that is publicly available.

Activity 1: Collation and review of existing evidence/information and gap analysis of first draft

The purpose of Step 1 is to collate and review available information on the intervention being documented. Examples of data sources to locate such information include: peer-reviewed publications, conference proceedings; publicly available reports, factsheets, annual reports and/or other internal publications; online sources including intervention or relevant government websites; financial/budget reports and/or personal communication with relevant program managers/organisations.

For interventions still in operation, current information may be gathered from websites and program reports. Where an intervention has ceased, historical information may be more challenging to locate. Information gaps revealed through this process may then require additional data to be collected.

Activity 2: Additional data collection to fill in the gaps

Planning for additional data collection may involve conducting interviews with stakeholders such as program managers/leaders, organisations or researchers who were involved during the formative development and decision-making of the intervention or implementation and scale-up process. Table 3 could be adapted to form an interview guide.

Activity 3: Review, report and disseminate findings

Following the additional data collection and information review, dissemination strategies for the completed SRG may include the production of the SRG as a case study or publishing the SRG findings in peer-reviewed journals, websites or external or internal reports to summarise key learning. SRG findings may also be fed back to the relevant stakeholders involved in the process to improve their understanding of the scale-up experience as a whole.

## 4. Discussion

The SRG developed in this study addresses a gap in the literature on implementation and scale-up in health promotion and chronic disease prevention by which researchers, policymakers and practitioners may capitalise on previous scale-up experience to guide future practice. With few articles describing the entire experience and lessons learned from the process of scaling up public health interventions under real-world conditions, there is a need to document these well and to share experiences and lessons to reduce duplication of ineffective methods and also to reduce the need to ‘reinvent the wheel’ [36]. Building on existing scale-up models, the SRG provides a structure to facilitate comprehensive capture of key information regarding the scale-up of an intervention in one document.

The SRG provides pilot-tested steps supporting its use and encourages the use of a range of credible information sources alongside multiple perspectives. Through the experience of pilot-testing the SRG it is recommended that the SRG should be completed by those with knowledge of the intervention and its scale-up, including program managers or members of the scale-up team with input from the delivery workforce, it is recognised that this may not always be possible. This is because the information to comprehensively complete the SRG may not be always be publicly available, and those with indirect experience and/or knowledge of the scale-up may be limited in their ability to reflect on the scale-up process. Nevertheless, pilot-testing the SRG on two scaled-up real-world interventions has demonstrated the utility of the SRG to comprehensively capture the information on the process of scale-up and adaptation of real-world interventions that is missing from current frameworks and guides.

Although the SRG was designed for reporting on the scale-up of interventions retrospectively, another advantage of this SRG, if used prospectively, is that it could potentially inform the stages of scaling up and reporting as they occur and could comprise part of the process evaluation of the intervention.

As the processes of scaling up of health promotion interventions vary considerably across different contexts and may follow a variety of pathways [10], documenting key steps and understanding the differences in delivery settings and the scale-up context will provide lessons for other similar interventions that are to be scaled [35]. Comprehensive documentation may reveal hitherto unrecognised strengths and weaknesses of different scale-up approach contexts under real-world conditions. Incorporating information on the process of adaptation on the intervention over time will further provide important contextual information to understand what may have led to intervention sustainment or cessation.

The development of a publicly available shared repository to store outputs of the SRG such as case studies would further enhance the value of the SRG [34]. Centralised web repositories of descriptions of interventions, such as the Evidence-Based Cancer Control Programs (EBCCP), have supported the selection of the effective interventions to address a range of chronic disease prevention programs [56]. While this and other online repositories in the USA [57] and the Netherlands [58] have also been developed to support public health decision-making broadly, none, to our knowledge, have been designed to capture and catalogue scale-up experiences. The SRG could serve as a structured guide to facilitate a standardised approach to developing scale-up case studies suitable for collation into an easily accessible repository [25,59].

### Limitations

The SRG was designed to be a pragmatic tool to reflect on and document experiences of scaling up interventions in the area of health promotion and chronic disease prevention, illustrated with physical activity and nutrition examples, though we believe its application could be broader to capture interventions across a broad spectrum of other health interventions. As a targeted approach to the literature was used, some aspects of the scale-up process could have been missed. To overcome this and to strengthen the validity of the SRG, we consulted with scale-up experts and pilot-tested the instrument with real-world interventions that were scaled up. However, we recognise that they too were limited in number. The next step would be more extensive testing in the real world and its practical application within the wider health and human services context.

Another acknowledged limitation of the SRG is that it may describe individual change interventions delivered at scale. It is meeting the expressed needs of real-world practitioners interested in scaling up their programs to reach wider populations, and the SRG will assist users in understanding why scale-up may not have worked. This is a similar research goal to our Intervention Scalability Assessment Tool (ISAT) [18], which is a tool for organisations and governments planning scaling-up interventions and provides guidance to assess the feasibility and readiness to go to scale. The SRG is a reactive tool and is not designed to assess different implementation approaches. It is standardised and therefore is not as in-depth as realist evaluations but will provide information that is comparable across scaled-up interventions.

Although our scale-up examples may be from single interventions, they can have real population-wide reach and can access hard-to-reach population groups [60,61]. Although this is not a whole-system intervention, it described programs that are major public health improvements on the myriad published programs of program effectiveness that only reach small volunteer samples. We recognise several limitations of this approach, including the effect sizes being attenuated when efficacious programs are delivered at scale [5,28]. Further, we acknowledge that we do not consider coding the upstream determinants of scale-up in the SRG, including social and political determinants, commercial determinants and influencers, and even transnational influences [62]. Nor will the SRG solve all the cross-sectoral partnerships needed to solve complex problems [63]. However, the upstream research to understand population health remains a complex contested field. Although there is a need for research integration between policy implementation research and implementation science [39], our evaluative approach provides a quite different and pragmatic approach to support health promotion and chronic disease prevention practice in real time. Our scale-up work and this SRG provides more immediate engagement to influence practice in real time.

## 5. Conclusions

The imperative to scale up effective interventions to achieve population-wide reach and impact has led to some advances in knowledge of how to scale up [11,64] in the area of health promotion and chronic disease prevention. However, in order to fully understand scale-up in real-world conditions, there needs to be a standardized approach to documenting the processes that underpin the development of the intervention, the intervention context, and its interaction with intervention implementation and from a variety of perspectives [35]. The SRG provides a structure and framework to reflect on and document scale-up experiences, which will improve comparisons and analysis of techniques, contexts and solutions to widespread dissemination of programs for population impact [35].

## Figures and Tables

**Table 1:** Common aspects of scale-up across frameworks.

Scale-Up Frameworks Reviewed	Intervention	Context	Decisions for Scaling Up	Service Delivery Organisation	Scale-Up Workforce	Scale-Up/ Implementation Process	Monitoring & Evaluation	Facilitators & Barriers	Sustainability
ExpandNet [2]	√	√	√	√	√	√	√	√	√
Simmons & Shiffman [14]	√	√	√	√	√	√	√	√	√
NSW Ministry of Health [6]	√	√	√	√	√	√	√	√	√
Yamey [11]	√	√		√	√	√	√		
Bhandari [47]	√	√		√	√	√	√	√	√
Cooley & Linn [7]	√	√	√	√	√	√	√	√	√
Fajans [12]	√	√	√	√	√	√	√	√	√

**Table 2:** Common aspects of scale-up.

Scale-Up Aspects	Explanation
1. **Intervention**	All frameworks discussed the need for the intervention (or ‘innovation’) to be adequately described. This included characteristics/attributes of the intervention considered for scale-up, including the target population, aims and components that are being scaled up.
2. **Context**	All frameworks acknowledged the importance of the context/environment in scaling up interventions. The context was termed loosely to include the political, social, economic, cultural or community at the macro and micro level at the time of scale-up that could influence scale-up.
3. **Decision making processes for scale-up**	Four of the six frameworks recognised the need for a mechanism for deciding if an intervention should be scaled up [2,6,7,14]. It was implied in the two remaining frameworks that the decision to scale-up had already been made; therefore, this aspect was not addressed. The decision-making process included assessments of scalability, availability of evidence, outcomes of pilot tests or trials and/or perceived relative advantage over other interventions.
4. **Delivery organisations**	Although the terminology varied, all frameworks reported that a key component of the scale-up process was the organisations or individuals responsible for delivery of the intervention, or those that are ‘expected to adopt or implement the intervention as part of the scale-up process’. This group was commonly referred to as ‘delivery organisations’ [6]; ‘user organisation’ [14]; ‘adopting community/organisation’ [7,11]. Important elements underpinning the ‘delivery organisation’ included a description of organisational capacity, governance and leadership, staffing, training considerations, resources and support as well as implementation strategies. For the purposes of consistency, the term ‘delivery organisations’ is used in this SRG.
5. **Scale-up workforce**	A variety of terms were used to describe the team or organisation overseeing or managing the scale-up process. This workforce is responsible for scale-up and may have been involved in development or testing of the intervention previously, and it is therefore termed an ‘originating organisation’ in some frameworks [6,7]. They may be described according to their setting (e.g., research, government, non-government) or they could be a single entity or a partnership or network of entities (or even individuals within different organisations). They may also be called the ‘resource organisation/team [14] or ‘implementers’ [11,47]. For consistency, we have used the term ‘scale-up workforce’. The distinction between the delivery organisation and scale-up workforce was widely recognised as essential; however, there may be overlap in terms of individuals in both groups [14].
6. **Scale-up process/strategy**	All frameworks discussed the importance of having a predetermined scale-up process (plan of steps and/or actions developed or undertaken to scale-up the intervention). For example, Yamey 2011 [11] described this as the ‘chosen delivery strategy’. For purposes of consistency, the term scale-up process has been selected for use within this SRG. Factors included in this scale-up process: - The vision/scope of scale-up—e.g., extent of expansion (size and/or geography) - Type of scale-up approach—horizontal, vertical scale-up or spontaneous - Type of scale-up design—centralised or decentralised - Costs of the scale-up and implementation - Resources/funding (including personnel) Additional plans and implementation strategies to be developed and incorporated into the scale-up process included strategies for: - Dissemination and advocacy such as champions or communications - Engagement and consultation with stakeholders - Adaptation/modification—the need to adapt or modify the intervention for scale-up and track subsequent changes in outcomes associated with the adaptations. Fajans [12] specifically indicated that this was a particular area of importance to cover when describing past scale-ups. - Maintenance of fidelity
7. **Monitoring and evaluation**	All frameworks indicated the need for comprehensive monitoring and evaluation activities as part of a scale-up to demonstrate the impact of the intervention. These activities include consistent data collection to monitor intervention and implementation progress to inform impacts and outcomes.
8. **Facilitators and barriers**	Potential attributes of success/facilitators of intervention transfer and scale-up across the areas along with the need to document lessons learned through scale-up related to: the intervention and/or its components, the nature and structure of the scale-up settings or the delivery organisations, the scale-up workforce, the changing political context and priorities and/or key actors influencing the process or the scale-up process employed.
9. **Sustainability**	Inherent in all frameworks as part of scale-up considerations was consideration of the sustainability of the intervention post-scale-up. Bhandari [47] described this as making sure there are ‘inbuilt provisions’ to the sustainability of the innovation. The impacts of scale-up on other interventions were also identified as being important to document [12].

**Table 3:** Scale-up Reflection Guide sections with a description of the types of information required.

**Section 1: SRG reporting details** **The Purpose of This Section Is to Document When This SRG Was Completed.**	
**1.1** **When the SRG was completed.** **1.2** **Persons completing the SRG and affiliations.** **1.3** **Main sources of information used for reporting this SRG.**	This section describes the recency of and background to the SRG completion. It includes information on when the SRG was completed, organisations completing this SRG and the data sources (e.g., peer-reviewed journal articles, government reports, websites) used to complete this SRG.
**Section 2: Intervention geographical location and scale-up approach** The purpose of this section is to record the geographical location and scale-up approach. Information on the intervention components and characteristics is to be captured in Section 4.	
**2.1** **Location of the intervention (i.e., geographical location/s).** **2.2** **Level of scale-up achieved (i.e., city, state or national scale).** **2.3** **Time period of scale-up.** **2.4** **Type of scale-up approach taken.** **2.5** **Current status of the intervention (active, no longer operational).**	This section describes where the intervention was scaled up, the magnitude of scale-up achieved in terms of size or geographical location and the scale-up approach taken. The latter includes (a) horizontal scaling up, which is often referred to as expansion or replication, sometimes as a regional introduction of an intervention followed by stepwise introduction in other regions; and (b) vertical scaling up, which involves simultaneous introduction of an intervention across the system, or (c) a combination of these approaches [6,48]. Current status should also be recorded, i.e., whether the intervention it is still active or when and why the intervention ceased.
**Section 3: Contextual and background information** The purpose of this section is to document historical and contextual information underpinning the need to scale up the intervention. If, over time, the nature of the target problem and or intervention context has changed, it may be useful to divide this section into two or more columns, as necessary, to highlight the changes during scale-up.	
**3.1** **Describe the nature of the problem.**	This section reports on the problem, rationale and perceived need for the intervention at the time of scale-up. Epidemiological data can be used to describe the magnitude of the problem.
**3.2** **Describe the strategic and political context.**	This section describes the political, strategic, environmental or policy contexts (including factors such as social and cultural acceptability, community values, needs of the population and funding structures) at the time of scale-up. Policy statements, strategic plans or related documents may be used to describe any influences from non-state state stakeholders (for example, industry or non-government sectors), if relevant. Any changes in context that have influenced the intervention and its scale-up should be documented.
**3.3** **Describe the strength of evidence of effectiveness that existed for the intervention.**	This section describes if/how the intervention had previously been demonstrated as effective (and effect size, if possible). Strength of evidence may be documented by using ‘Levels of evidence’ frameworks [49,50], including systematic reviews or RCTs in this section.
**3.4** **Describe the decision-making process including documenting the key actors and their roles along with other factors that may have influenced this process.**	This section documents the steps in the scale-up decision-making process, and key actors and their organisational roles. Where possible, report on the factors that influence decisions to scale up interventions
**Section 4: The intervention** The purpose of this section is to document key information about the intervention, including its purpose, target audience and a description of the key elements. If the current scaled-up intervention has changed from its original form or has been scaled up incrementally, it may be useful to divide this section into two or more columns, as necessary, to highlight the changes during scale-up. An example of how this can be done is provided in the examples in Supplementary File S2.	
**4.1** **Aims/objectives.**	This section describes what the intervention is attempting to achieve in relation to the target population and addressing the problem (Section 3.1).
**4.2** **Target population.**	
**4.3** **Key intervention elements/components.**	This section describes the intervention, including underpinning theories and/or principles, along with identified ‘core’ and/or ‘flexible’ components of the intervention and its modes of delivery. Where multiple components exist, each component should be described and classified as either core and/or flexible components.
**4.4** **Describe any modifications or adaptations required to the intervention components to enable scale-up.**	This section provides detailed descriptions of modifications and adaptations to the intervention itself, i.e., its components for scale-up and whether any additional research/testing was conducted on the adapted components. Where available, identify the stakeholders responsible for making any modification and adaptation and their reasons for making these modifications and adaptations.
**Section 5: Intervention costs, funding and partnership arrangements** The purpose of this section is to document the costs and funding arrangements for the intervention being described. If, over time, the funding provision or costs have changed, it may be useful to divide this section into two or more columns, as necessary, to highlight the changes during scale-up. An example of how this can be done is provided in the examples in the Supplementary File S2.	
**5.1** **Describe the cost(s) associated with the set-up/scale-up or delivery of the intervention.**	This section provides sources of funding and funding arrangements or partnerships, as well as costs associated with (a) intervention delivery (materials, infrastructure, workforce), (b) any other costs associated with the scale-up process, or c) costs for participants to participate (if any). Resources may be provided through one organisation or through a partnership across multiple organisations. Changes in funding arrangements or in overall costs over time should be reported. It would also be helpful to document the nature of the funding, e.g., fixed term (and if so, for how long) or ongoing.
**5.2** **Describe the source of funding and any funding arrangements underpinning it (e.g., co-funding, public/private). Describe any changes to this funding source or arrangements over time.**	
**Section 6: The scale-up setting and delivery** The purpose of this section is to document information on the setting in which the intervention was scaled up as well as the delivery organisation and/or workforce employed. If, over time, the setting has changed, it may be useful to divide this section into two or more columns, as necessary, to highlight the changes during scale-up. An example of how this can be done is provided in the examples in Supplementary File S2.	
**6.1** **Describe the setting the intervention was scaled up in.**	This section documents the setting in which the intervention was scaled up, such as ‘Education’ sector, including pre-school or childcare settings, ‘Workplaces’, and ‘Health care’ settings. If multiple settings are used, this should be documented.
**6.2** **Describe the delivery organisation and/or workforce.**	This section describes the delivery organisation(s) and workforce. The delivery organisation and/or workforce refers to organisations or personnel responsible for delivering the intervention, e.g., if the intervention was delivered in a school setting, the delivery organisation may be the school while the delivery workforce may be teachers within the school. Any external organisations/consultants used as delivery agents should also be documented.
**6.3** **Describe any partnerships that were formed to help support or manage the delivery of the intervention.**	This section describes any partnerships formed to build capacity, provide resources or deliver or manage components of the scaled-up intervention. These partners may be internal or external to the delivery organisation or scale-up workforce, for example, food or sports promotion bodies or even commercial partners.
**6.4** **Describe any implementation strategies that were used to aid the implementation of the intervention in their delivery settings.**	This section describes any additional implementation strategies or actions developed to facilitate implementation in different settings. These additional strategies may include strategies for communication and engagement, or resources and training [51,52].
**6.5** **Describe any modifications or adaptations required at the delivery setting/organisation/workforce level in order to scale-up the intervention.**	This section describes any modifications (if any) made to the delivery process in order to scale up. Examples include changing or redefining goals, changing funding support structures or making modifications in response to feedback from the delivery settings/workforce themselves. Describing the process of adaptation, reasons for adaptations, and who made them, would be useful.
**Section 7: The scale-up process** The purpose of this section is to document the scale-up process along with the scale-up workforce, resources available for managing and assisting with the scale-up process, and any evidence generated through this process.	
**7.1** **Describe the process undertaken to scale-up the intervention.**	This section documents the scale-up process in detail. There are numerous process models that provide guidance on how to scale up interventions [6,7,13,14,53,54] but in practice, the scale-up process may be different for every intervention [10]. This section outlines key steps and/or activities undertaken, the stages and timeframe, and the key actors that were involved in each step/activity. Visual representations as timelines or flow diagrams may be useful to illustrate the process.
**7.2** **Describe the ‘scale-up workforce’ used to support the scale-up process.**	The ‘scale-up’ workforce is the organisation or team involved in overseeing or facilitating the implementation at scale (e.g., government, non-government, philanthropic organisations) and may have also been involved in the development and testing of the innovation [14]. This section describes the existence, structure and/or roles of the scale-up team and individual members and how it was formed. For example, was there a specific team established to implement the scale-up process? Were the members recruited externally for this purpose or were they existing personnel? Their roles may include coordinating the scale-up process across multiple agencies, providing or building capacity and infrastructure for the scale-up process and/or delivering components of the intervention itself. How were they structured? For example, they are often (but not always) part of the organisation responsible for scale-up and may be centralised (located in one area) or decentralised (located across multiple location/sites).
**7.3** **Describe any partnerships with other organisations to help support or manage the scale-up of the intervention.**	Partnerships/collaborations may build capacity or resources or improve funding stability; these partnerships may support, manage or even accelerate the scale-up process. This section differs from 6.3 in that the partnerships here are partnerships that support the scale-up process and not just the intervention delivery.
**7.4** **Describe the governance/leadership and management structure directing the scale-up process.**	This section describes governance of the scale-up process. For example, was there a specific management/governing committee? Who was on such a committee, and what were their roles and responsibilities? What feedback is available about their perceived effectiveness in supporting the scale-up process?
**7.5** **Describe any strategies that were used for scale-up (including communication strategies and advocacy).**	This section describes additional implementation strategies or actions developed to facilitate the scale-up process not already covered in Section 7.1 or Section 6.4, [15,51,52,55]. Additional strategies may include those for communication and engagement, policy dialogues, stakeholder management, change management, technological infrastructure [51,52]. This section describes any such implementation strategies along with their effect on the scale-up process.
**7.6** **Describe any barriers or facilitators in scaling up the intervention and strategies used to overcome barriers.**	This section reports on any factors that contributed to the success or challenged the implementation of the intervention at scale. They may relate to the intervention and/or its components, the nature and structure of the scale-up settings or the delivery organisations, or even the scale-up workforce, changing political context and priorities, key actors influencing the process or the scale-up process employed [9,14,19]. Useful details may include whether the facilitators and barriers were known prior to scale-up or discovered as a result of implementation. This section should report any mitigation actions or strategies taken to address any challenges.
**7.7** **Describe any strategies that have been developed to ensure the intervention’s sustainability.**	This section covers any pre scale-up planning undertaken to guide the long term direction, goals and strategies of the intervention in terms of resourcing, funding arrangements or stakeholder involvement, and where known, the impact of planning on intervention sustainability.
**Section 8: Evidence of effectiveness and long-term outcomes** The purpose of this section is to describe any research and/or evaluation activities conducted during scale-up or post-scale-up to determine the impact, outcome and/or effectiveness of the intervention. Research and/or evaluation into processes and implementation should also be documented. In this section, descriptions of longer-term outcomes resulting from the scale-up should also documented.	
**8.1** **Describe the evidence generated during scale-up or post-scale-up on the implementation, impact and/or outcome of the intervention at-scale.**	This section reports on (a) monitoring and/or evaluation activities (such as formative, process, impact or outcome evaluations) conducted during or post-scale-up, and (b) the results of such monitoring and evaluation activities (for example, what adaptations/changes were introduced as a result of evaluation outcomes).
**8.2** **Describe if any other interventions were de-implemented or modified as a result of the intervention that was scaled up.**	This section should describe the impact of the scaled-up intervention on other related interventions within the setting or context. If the scaled-up intervention had an impact on other deliveries of other programs, this should be documented. For example, as a result of the intervention that was scaled up, did it result in other activities being de-implemented? Did it result in changes to other services delivered?
**8.3** **Describe the sustainability of the intervention post-scale-up**	This section documents the outcomes following scale-up, particularly if the intervention was sustained over time, and whether the intervention was taken up as policy. For example, was it scaled up in other regions or countries?

## Data Availability

No new data were created or analyzed in this study. Data sharing is not applicable to this article.

## Supplementary Materials

The following supporting information can be downloaded at: https://www.mdpi.com/article/10.3390/ijerph20116014/s1, Figure S1: Literature Review results; Table S1: Summary of scale-up process frameworks identified and inclusion/exclusion status; Table S2: Scale-up Reflection Guide rationale; Case studies S1: Two completed examples of scale-up using the Scale-up Reflection Guide. References [65,66,67,68,69,70,71,72,73,74,75,76,77,78,79] are mentioned in Supplementary Materials.

## References

[1] Milat A., King L., Bauman A., Redman S. (2013). The concept of scalability: Increasing the scale and potential adoption of health promotion interventions into policy and practice. Health Promot. Int..

[2] World Health Organisation Nine Steps for Developing a Scaling-Up Strategy, 2010. http://www.who.int/reproductivehealth/publications/strategic_approach/9789241500319/en/.

[3] Naylor D., Girard F., Mintz J., Fraser N., Jenkins T., Power C. Unleashing Innovation: Excellent Healthcare for Canada: Report of the Advisory Panel on Healthcare Innovation, 2015. https://healthycanadians.gc.ca/publications/health-system-systeme-sante/report-healthcare-innovation-rapport-soins/alt/report-healthcare-innovation-rapport-soins-eng.pdf.

[4] Ben Charif A., Zomahoun H., LeBlanc A., Langlois L., Wolfenden L., Yoong S., Williams C.M., Lépine R., Légaré F. (2017). Effective strategies for scaling up evidence-based practices in primary care: A systematic review. Implement. Sci..

[5] Lane C., McCrabb S., Nathan N., Naylor P.-J., Bauman A., Milat A., Lum M., Sutherland R., Byaruhanga J., Wolfenden L. (2021). How effective are physical activity interventions when they are scaled-up: A systematic review. Int. J. Behav. Nutr. Phys. Act..

[6] Milat A., Newson R., King L. Increasing the Scale of Population Health Interventions: A Guide.

[7] Cooley L., Kohl R., Ved R. (2016). Scaling Up—From Vision to Large Scale Change: A Management Framework for Practitioners.

[8] Milat A.J., King L., Newson R., Wolfenden L., Rissel C., Bauman A., Redman S. (2014). Increasing the scale and adoption of population health interventions: Experiences and perspectives of policy makers, practitioners, and researchers. Health Res. Policy Syst..

[9] Norton W., Mittman B. (2010). Scaling-Up Health Promotion/Disease Prevention Programs in Community Settings: Barriers, Facilitators and Initial Recommendations.

[10] Indig D., Lee K., Grunseit A., Milat A., Bauman A. (2018). Pathways for scaling up public health interventions. BMC Public Health.

[11] Yamey G. (2011). Scaling up global health interventions: A proposed framework for success. PLoS Med. Public Libr. Sci..

[12] Fajans P., Ghiron L., Kohl R., Simmons R. (2007). 20 Questions for developing a scaling up case study. Manag. Syst. Int..

[13] World Health Organisation ExpandNet Pracical Guidance for Scaling Up Health Service Innovations, 2009. http://apps.who.int/iris/bitstream/handle/10665/44180/9789241598521_eng.pdf;jsessionid=F4BCDBE30D12C3EE031541604E8CE7BE?sequence=1.

[14] Simmons R., Shiffman J., Simmons R., Ghiron L. (2007). Scaling up health service innovations: A framework for action. Scaling Up Health Service Delivery.

[15] Damschroder L., Aron D., Keith R., Kirsh S., Alexander J., Lowery J. (2009). Fostering implementation of health services research findings into practice: A consolidated framework for advancing implementation science. Implement. Sci..

[16] Durlak J., DuPre E. (2008). Implementation matters: A review of research on the influence of implementation on program outcomes and the factors affecting implementation. Am. J. Community Psychol..

[17] Koorts H., Eakin E., Estabrooks P., Timperio A., Salmon J., Bauman A. (2018). Implementation and scale up of population physical activity interventions for clinical and community settings: The PRACTIS guide. Int. J. Behav. Nutr. Phys. Act..

[18] Milat A., Lee K., Conte K., Grunseit A., Wolfenden L., van Nassau F., Orr N., Sreeram P., Bauman A. (2020). Intervention Scalability Assessment Tool: A decision support tool for health policy makers and implementers. Health Res. Policy Syst..

[19] Milat A., Bauman A., Redman S. (2015). Narrative review of models and success factors for scaling up public health interventions. Implement. Sci..

[20] Bulthuis S., Kok M., Raven J., Dieleman M. (2020). Factors influencing the scale-up of public health interventions in low- and middle-income countries: A qualitative systematic literature review. Health Policy Plan..

[21] Webster J., Chandramohan D., Hanson K. (2010). Methods for evaluating delivery systems for scaling-up malaria control intervention. BMC Health Serv. Res..

[22] Milat A., Bauman A., Redman S. (2015). A narrative review of research impact assessment models and methods. Health Res. Policy Syst..

[23] Bégin H., Eggertson L., Macdonald N. (2009). A country of perpetual pilot projects. Can. Med. Assoc. J..

[24] Eaton J., McCay L., Semrau M., Chatterjee S., Baingana F., Araya R., Ntulo C., Thornicroft G., Saxena S. (2011). Scale up of services for mental health in low-income and middle-income countries. Lancet.

[25] Reis R., Salvo D., Ogilvie D., Lambert E., Goenka S., Brownson R. (2016). Scaling up physical activity interventions worldwide: Stepping up to larger and smarter approaches to get people moving. Lancet.

[26] Albury D., Beresford T., Dew S., Horton T., Illingworth J., Langford K. Against the Odds: Successfully Scaling Innovation in the NHS. London (UK) Innovation Unit and The Health Foundation, 2018. https://www.innovationunit.org/wp-content/uploads/Against-the-Odds-Innovation-Unit-Health-Foundation.pdf.

[27] Hawn C. Going Big: How Major Providers Scale Up Their Best Ideas. Oakland, United States: California Health Care Foundation, 2012. https://www.chcf.org/wp-content/uploads/2017/12/PDF-GoingBigProvidersScaleUpIdeas.pdf.

[28] Sutherland R., Campbell E., McLaughlin M., Nathan N., Wolfenden L., Lubans D.R., Morgan P.J., Gillham K., Oldmeadow C., Searles A. (2020). Scale-up of the Physical Activity 4 Everyone (PA4E1) intervention in secondary schools: 12-month implementation outcomes from a cluster randomized controlled trial. Int. J. Behav. Nutr. Phys. Act..

[29] Begg C., Cho M., Eastwood S., Horton R., Moher D., Olkin I., Pitkin R., Rennie D., Schulz K.F., Simel D. (1996). Improving the Quality of Reporting of Randomized Controlled Trials: The CONSORT Statement. JAMA.

[30] Moher D., Schulz K., Altman D. (2001). The CONSORT statement: Revised recommendations for improving the quality of reports of parallel-group randomized trials. JAMA.

[31] von Elm E., Altman D.G., Egger M., Pocock S.J., Gøtzsche P.C., Vandenbroucke J.P. (2008). The Strengthening the Reporting of Observational Studies in Epidemiology (STROBE) statement: Guidelines for reporting observational studies. J Clin Epidemiol.

[32] Tong A., Sainsbury P., Craig J. (2007). Consolidated criteria for reporting qualitative research (COREQ): A 32-item checklist for interviews and focus groups. Int. J. Qual. Health Care.

[33] Albrecht L., Archibald M., Arseneau D., Scott S. (2013). Development of a checklist to assess the quality of reporting of knowledge translation interventions using the Workgroup for Intervention Development and Evaluation Research (WIDER) recommendations. Implement. Sci..

[34] Kruk M.E., Yamey G., Angell S.Y., Beith A., Cotlear D., Guanais F., Jacobs L., Saxenian H., Victora C., Goosby E. (2016). Transforming Global Health by Improving the Science of Scale-Up. PLoS Biol..

[35] Greenhalgh T., Papoutsi C. (2019). Spreading and scaling up innovation and improvement. BMJ Clin. Res..

[36] Whitworth J., Sewankambo N., Snewin V. (2010). Improving Implementation: Building Research Capacity in Maternal, Neonatal, and Child Health in Africa. PLoS Med..

[37] Solomon J. (2006). Case Studies: Why are they important?. Nat. Clin. Pract. Cardiovasc. Med..

[38] Crowe S., Cresswell K., Robertson A., Huby G., Avery A., Sheikh A. (2011). The case study approach. BMC Med. Res. Methodol..

[39] Nilsen P. (2015). Making sense of implementation theories, models and frameworks. Implement. Sci..

[40] Canadian Agency for Drugs and Technology in Health Grey Matters: A Practical Tool for Searching Health-Related Grey Literature Ottawa 2018 [Updated 2019]. https://www.cadth.ca/resources/finding-evidence.

[41] McKay H., Naylor P.-J., Lau E., Gray S.M., Wolfenden L., Milat A., Bauman A., Race D., Nettlefold L., Sims-Gould J. (2019). Implementation and scale-up of physical activity and behavioural nutrition interventions: An evaluation roadmap. Int. J. Behav. Nutr. Phys. Act..

[42] Shelton R., Cooper B., Stirman S. (2018). The Sustainability of Evidence-Based Interventions and Practices in Public Health and Health Care. Annu. Rev. Public Health.

[43] Stirman S., Kimberly J., Cook N., Calloway A., Castro F., Charns M. (2012). The sustainability of new programs and innovations: A review of the empirical literature and recommendations for future research. Implement. Sci..

[44] Stirman S., Baumann A., Miller C. (2019). The FRAME: An expanded framework for reporting adaptations and modifications to evidence-based interventions. Implement. Sci..

[45] Chambers D., Norton W. (2016). The Adaptome: Advancing the Science of Intervention Adaptation. Am. J. Prev. Med..

[46] Chambers D., Glasgow R., Stange K. (2013). The dynamic sustainability framework: Addressing the paradox of sustainment amid ongoing change. Implement. Sci..

[47] Bhandari N., Kabir A., Salam M. (2008). Mainstreaming nutrition into maternal and child health programmes: Scaling up of exclusive breastfeeding. Matern. Child Nutr..

[48] World Health Organisation Scaling Up Projects and Initiatives for Better Health: From Concepts to Practice. Denmark, 2016. https://www.euro.who.int/en/publications/abstracts/scaling-up-projects-and-initiatives-for-better-health-from-concepts-to-practice-2016.

[49] Evans D. (2003). Hierarchy of evidence: A framework for ranking evidence evaluating healthcare interventions. J. Clin. Nurs..

[50] Merlin T., Weston A., Tooher R. (2009). Extending an evidence hierarchy to include topics other than treatment: Revising the Australian ‘levels of evidence’. BMC Med Res Methodol.

[51] Powell B.J., Waltz T.J., Chinman M.J., Damschroder L.J., Smith J.L., Matthieu M.M., Proctor E.K., Kirchner J. (2015). A refined compilation of implementation strategies: Results from the Expert Recommendations for Implementing Change (ERIC) project. Implement. Sci..

[52] Leeman J., Birken S., Powell B., Rohweder C., Shea C. (2017). Beyond “implementation strategies”: Classifying the full range of strategies used in implementation science and practice. Implement. Sci..

[53] Spicer N., Bhattacharya D., Dimka R., Fanta F., Mangham-Jefferies L., Schellenberg J., Tamire-Woldemariam A., Walt G., Wickremasinghe D. (2014). ‘Scaling-up is a craft not a science’: Catalysing scale-up of health innovations in Ethiopia, India and Nigeria. Soc. Sci. Med..

[54] Barker P., Reid A., Schall M. (2016). A framework for scaling up health interventions: Lessons from large-scale improvement initiatives in Africa. Implement. Sci..

[55] Proctor E., Luke D., Calhoun A., McMillen C., Brownson R., McCrary S., Padek M. (2015). Sustainability of evidence-based healthcare: Research agenda, methodological advances, and infrastructure support. Implement. Sci..

[56] National Cancer Institute Evidenced-Based Cancer Control Programs USA: U.S. Department of Health and Human Services. https://ebccp.cancercontrol.cancer.gov/index.do.

[57] UNC Center for Health Promotion and Disease Prevention SNAP-Ed Toolkit: Obesity Prevention Interventions and Evaluation Framework 2020 [updated 31 July 2020]. https://snapedtoolkit.org/.

[58] Brug J., van Dale D., Lanting L., Kremers S., Veenhof C., Leurs M., van Yperen T., Kok G. (2010). Towards evidence-based, quality-controlled health promotion: The Dutch recognition system for health promotion interventions. Health Educ. Res..

[59] Pinnock H., Epiphaniou E., Pearce G., Parke H., Greenhalgh T., Sheikh A., Griffiths C.J., Taylor S.J.C. (2015). Implementing supported self-management for asthma: A systematic review and suggested hierarchy of evidence of implementation studies. BMC Med.

[60] Fehily C., Bartlem K., Wiggers J., Wye P., Clancy R., Castle D., Wutzke S., Rissel C., Wilson A., McCombie P. (2017). Evaluating the effectiveness of a healthy lifestyle clinician in addressing the chronic disease risk behaviours of community mental health clients: Study protocol for a randomised controlled trial. Trials.

[61] Cranney L., Wen L.M., Xu H., Tam N., Whelan A., Hua M., Ahmed N. (2018). Formative research to promote the Get Healthy Information and Coaching Service (GHS) in the Australian-Chinese community. Aust. J. Prim. Health.

[62] Hawkins B., Holden C., Mackinder S. (2020). Policy Transfer in the Context of Multi-level Governance. The Battle for Standardised Cigarette Packaging in Europe: Multi-Level Governance, Policy Transfer and the Integrated Strategy of the Global Tobacco Industry.

[63] De Leeuw E., Peters D. (2014). Nine questions to guide development and implementation of Health in All Policies. Health Promot. Int..

[64] Smith J., de Graft-Johnson J., Zyaee P., Ricca J., Fullerton J. (2015). Scaling up high-impact interventions: How is it done?. Int. J. Gynaecol. Obstet..

[65] Cooley L., Linn J. (2014). Taking Innovations to Scale: Methods, Applications and Lessons.

[66] Kohl R., Cooley L. (2003). Scaling Up—A Conceptual and Operational Framework.

[67] Pérez-Escamilla R., Curry L., Minhas D., Taylor L., Bradley E. (2012). Scaling up of breastfeeding promotion programs in low-and middle-income countries: The “breastfeeding gear” model. Adv. Nutr. Int. Rev. J..

[68] Wandersman A., Duffy J., Flaspohler P., Noonan R., Lubell K., Stillman L., Blachman M., Dunville R., Saul J. (2008). Bridging the gap between prevention research and practice: The interactive systems framework for dissemination and implementation. Am. J. Community Psychol..

[69] Edwards N., Barker P.M. (2014). The importance of context in implementation research. J. Acquir. Immune Defic. Syndr..

[70] Pelletier D., Corsi A., Hoey L., Faillace S., Houston R. (2011). The Program Assessment Guide: An approach for structuring contextual knowledge and experience to improve the design, delivery, and effectiveness of nutrition interventions. J. Nutr..

[71] Hirschhorn L.R., Talbot J.R., Irwin A.C., A May M., Dhavan N., Shady R., Ellner A.L., Weintraub R.L. (2013). From scaling up to sustainability in HIV: Potential lessons for moving forward. Glob. Health.

[72] Greenhalgh T., Wherton J., Papoutsi C., Lynch J., Hughes G., A’Court C., Hinder S., Fahy N., Procter R., Shaw S. (2017). Beyond Adoption: A New Framework for Theorizing and Evaluating Nonadoption, Abandonment, and Challenges to the Scale-Up, Spread, and Sustainability of Health and Care Technologies. J. Med. Internet Res..

[73] Bezanson K., Isenman P. (2010). Scaling up nutrition: A framework for action. Food Nutr. Bull..

[74] Lobo R., Petrich M., Burns S.K. (2014). Supporting health promotion practitioners to undertake evaluation for program development. BMC Public Health.

[75] Bernal G., Domenech Rodiguez M. (2012). Cultural Adaptations: Tools for Evidence-Based Practice with Diverse Populations.

[76] Schell S.F., Luke D.A., Schooley M.W., Elliott M.B., Herbers S.H., Mueller N.B., Bunger A.C. (2013). Public health program capacity for sustainability: A new framework. Implement. Sci..

[77] Scheirer M.A. (2013). Linking sustainability research to intervention types. Am. J. Public Health.

[78] Norton W.E., Chambers D.A. (2020). Unpacking the complexities of de-implementing inappropriate health interventions. Implement. Sci..

[79] Palinkas L.A., Chou C.-P., Spear S.E., Mendon S.J., Villamar J., Brown C.H. (2020). Measurement of sustainment of prevention programs and initiatives: The sustainment measurement system scale. Implement. Sci..

