# Supplementary material for: Development and Application of the Scale-Up Reflection Guide (SRG)
Source: Int J Environ Res Public Health. 2023 May 31;20(11):6014. doi: 10.3390/ijerph20116014 (PMC10253157; doi:10.3390/ijerph20116014)

Supplementary files

Figure S1: Literature Review results

Table S1: Summary of scale-up process frameworks identified and inclusion/exclusion status

Table S2: Scale-up Reflection Guide rationale

Case studies S1: Two completed examples of scale-up using the Scale-up Reflection Guide

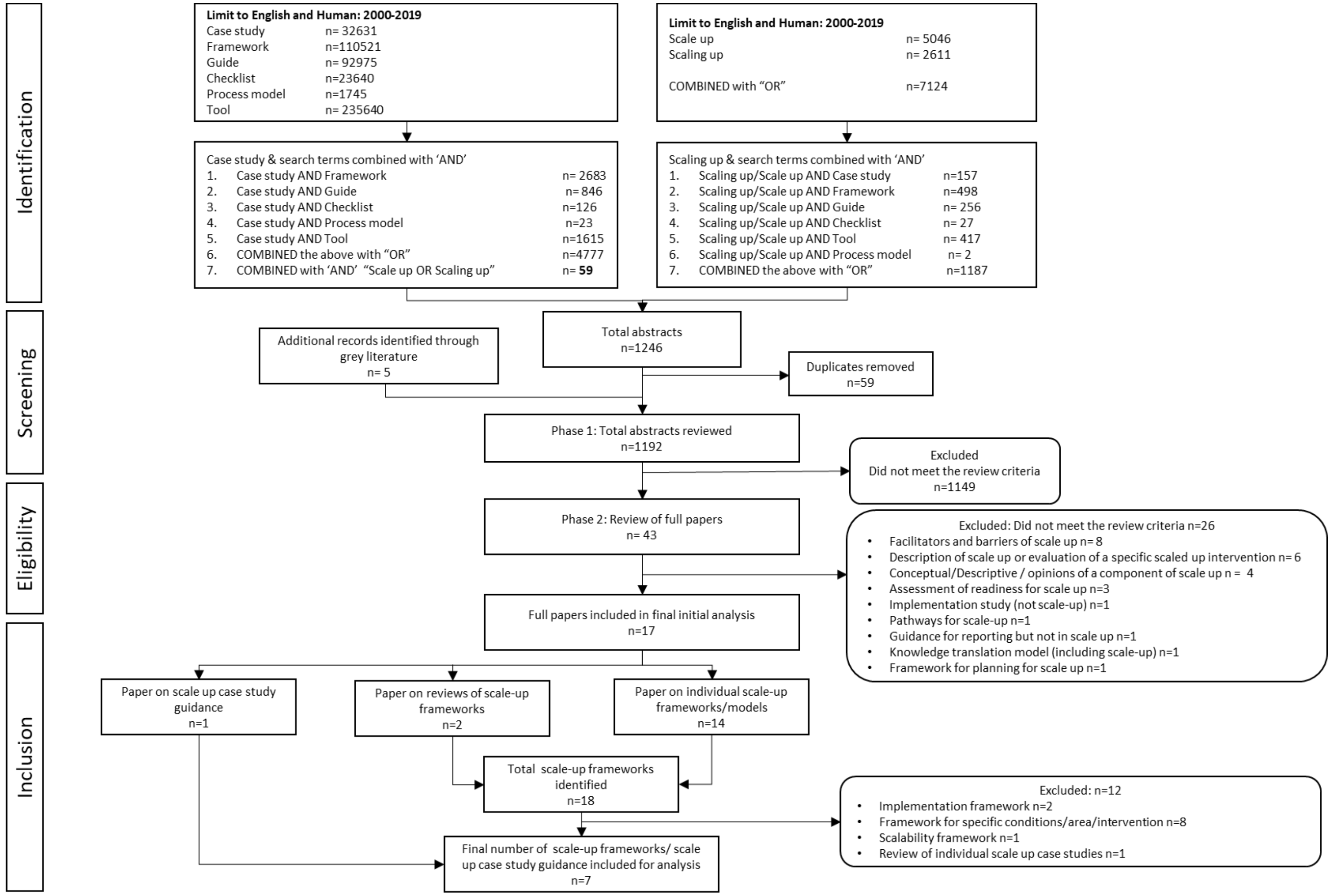

Figure S1: Literature Review results.

Table S1: Summary of scale-up process frameworks identified and inclusion/exclusion status

Scale-up process frameworks identified	Scale-up focus	Identified in literature search	REVIEW BY Milat et al 2015 (19)	REVIEW BY McKay et al 2019 (41)	Inclusion (reason excluded)
**1. ExpandNet (WHO) (2)**	General/ non-specific scale-up	√	√	√	Yes
**2. Yamey 2011 (11)**	General/ non-specific scale-up	√	√	√	Yes
**3. NSW Ministry of Health (6)**	General/ non-specific scale-up	√	√	√	Yes
**4. Bhandahari 2008 (47)**	Health Promotion/ General	√	√		Yes
**5. Cooley et al (7, 65, 66)**	General/ non-specific scale-up	√	√		Yes
**6. Simmons & Shiffman (14)**	General/ non-specific scale-up	√	√	√	Yes
**7. Fajans (12) (Scale up case study guide)**	General/ non-specific scale-up	√			Yes
**8. Koorts et al (17)**	Physical Activity	√			No (implementation framework)
**9. Perez-Escamilla et al (67)**	Breastfeeding		√		No (specific condition)
**10. Wandersman (68)**	Implementation framework			√	No (implementation framework)
**11. Reis et al (67)**	Physical Activity			√	No (review of scale-up case studies)
**12. Spicer et al (53)**	Maternal and newborn health	√			No (specific intervention)
**13. Barker et al (54)**	Global health	√			No (specific condition(s))

**14. Edwards & Barker (69)**	HIV	√			No (specific condition)
**15. Pelletier et al (70)**	Nutrition	√			No (specific condition)
**16. Milat et al (1)**	Scalability assessment		√	√	No (scalability assessment only)
**17. Hirschhorn (71)**	HIV	√			No (specific condition)
**18. Greenhalgh et al (72)**	Health technology	√			No (specific area)
**19. Bezanson & Isenman (73)**	Nutrition	√			No (specific area)

Table S2. Scale-up Reflection Guide rationale.

This table provides the rationale from the literature as to why each aspect is critical for documenting in the SRG.

**Section/Questions**	**Rationale**
**Section 1: SRG reporting details**<br>The purpose of this section is to document when and how this SRG was completed.	
1.1 When the SRG was completed.<br>1.2 Persons completing the SRG and affiliations.<br>1.3 Main sources of information used for reporting this SRG	The types of the information and sources used as well as the background of the person/organisations completing the SRG provides important context for the reader to determine the currency and credibility of the SRG.
**Section 2: Intervention geographical location and scale-up approach**<br>The purpose of this section is to record the geographical location and scale-up approach of the intervention. Information on the intervention components and characteristics is to be captured in Section 4.	
2.1 Location of the intervention (i.e. geographical location/s).<br>2.2 Level of scale-up achieved (i.e. city, state or national scale).<br>2.3 Time period of scale-up.<br>2.4 Type of scale-up approach taken.<br>2.5 Current status of the intervention (active, no longer operational).	The geographical location and level and type of scale-up achieved provides environmental context for the intervention's potential generalisability (6, 48).
**Section 3: Contextual and background information**<br>The purpose of this section is to document historical and contextual information underpinning the need to scale-up the intervention. If, over time the nature of the target problem and or intervention context changed, it may be useful to divide this section into two or more columns as necessary to highlight the changes during scale-up.	
3.1 Describe the nature of the problem.	The nature of the problem and the perceived need for the intervention at the time of scale-up is critical for understanding the historical context of the problem and motivations for scale-up, more so if the context at the time of reporting has changed (12).

3.2 Describe the strategic and political context.	Strategic or political priorities at various levels have been cited as success factors or even barriers in the scale-up of many programs (7, 11). Describing these factors which may include social and cultural acceptability, community values, needs of the population and funding structures at the time of scale-up may offer insights into why some scale-up experiences are more successful in some contexts and not others (11). The political environment in particular may have changed over time and can influence the program's resources, funding stability or acceptance (12, 55).
3.3 Describe the strength of evidence of effectiveness that existed for the intervention.	The research evidence generated to test the efficacy or demonstrate the effectiveness of the intervention (1, 6, 7, 48) provides the evidence base for the intervention, how it was developed and piloted (14, 74)
3.4 Describe the decision-making process including documenting the key actors and their roles along with other factors that may have influenced this process.	Understanding the factors that influence decisions to scale-up interventions as well as the decision-making process itself is infrequently reported (1, 6, 8). Decisions to scale-up an intervention are often influenced by a range of factors, including the intervention's feasibility, acceptability and adaptability as well as key actors that contributed to final decision to scale-up (8). As an example, it has been reported that researchers generally may be involved in formulating scale-up recommendations, however, ultimately decisions are made by policy-makers or politicians within government agencies over which they have little control, while policy-makers on the other had are often guided by the political and strategic context and their reliance on research evidence for decision-making for scale-up is sporadic at best (8).
**Section 4: The intervention**<br>The purpose of this section is to document key information about the intervention including its purpose, target audience and a description of the key elements.	
4.1 Aims/objectives.	Describing the characteristics of the intervention, its aim and target population is important for explaining the need for the scaling up the intervention to address the problem previously described (Section 3.1). The intervention has to provide some benefit and fit with the needs of the target population if it is to be adopted and sustained over the longer term (16).
4.2 Target population.	
4.3 Key intervention elements/components.	A description of the intervention, including the underpinning theories or principles, along with any 'core' and/or 'flexible' components and methods of delivery is helpful for

	understanding how the intervention works in practice. Distinguishing the core and/or flexible intervention components is useful as it highlights the components which are potentially modifiable for future adaptation of the intervention [11].
4.4 Describe any modifications or adaptations required to the intervention components to enable scale-up.	Rarely is there a 'one size fits all' intervention that can be implemented in all settings or contexts without the need for modification or adaptation. This need often arises due to a incompatibilities between the original intervention and contextual factors such as the delivery system, target population (e.g. cultural or demographic differences) or available infrastructure or workforce (75). It is important to identify if incompatibilities exist in order to create modifications or adapt elements of the intervention to facilitate a better fit (12, 45). Adaptations can be planned or unplanned, motivated by social-technological, political environmental factors, internal organisational factors or simply to improve the intervention reach (15, 44). Understanding this process is critical for advancing our understanding of the science of adaptation.
**Section 5: Intervention costs, funding and partnership arrangements**<br>The purpose of this section is to document the costs, funding arrangements for the intervention being described.	
5.1 Describe the cost(s) associated with the set up/ scale-up or delivery of the intervention.	Key sources of funding and funding arrangements differ across interventions and different strategies may be implemented at the delivery stage or the scale-up process which may ultimately impact its success and sustainment (19). Adequately recording the level and types of funding support as well as costs of intervention delivery along with any changes over time will help researchers understand more about how interventions are sustained (76).
5.2 Describe the source of funding and any funding arrangements underpinning it (e.g., co-funding, public private). Describe any changes to this funding source or arrangements over time.	
**Section 6: The scale-up 'delivery' process**<br>The purpose of this section is to document information on the setting in which the intervention was scaled up in as well as the delivery organisation and/or workforce employed.	
6.1 Describe the setting the intervention was scaled up in.	Different interventions features may be required for different settings and the setting in which the intervention is delivered will inform others as to its potential replicability. The

	intervention setting may also affect the sustainability of an intervention (42) and therefore is important to document.
6.2 Describe the delivery organisation and/or workforce.	Detailed descriptions of the delivery organisation, workforce, their role is important for understanding the scale- process, particularly in identifying success factors and/or barriers to scale-up. Where the intervention is well aligned with the goals and philosophy of the delivery organisations, it often leads to greater scale-up success (14, 16) while a misalignment along with insufficient resources, internal staff turnover, inadequate support or poor communication and engagement can have a negative impact on scale-up (19). Resistance to new policies and processes, poor support within the organisation (lack of someone to champion the intervention) can also pose as a barrier to scale-up (12, 19). Understanding these factors through detailed documentation will help guide future scale-up attempts in similar settings with similar delivery workforce.
6.3 Describe any partnerships that were formed to help support or manage the delivery of the intervention	Intervention delivery often takes place in a broader context and partnerships/collaborations between multiple partners as a strategy to build capacity, resource or improve funding stability may have been formed to support, manage or even accelerate the scale-up process. Such strategies have been noted to be helpful in facilitating more successful outcomes (77) and documenting the details of such partnerships if occurred, will promote greater in-depth understanding how it may work and the factors is required for coordinating different administrative structures and how these partnerships work in practice (42).
6.4 Describe any implementation-strategies that were used to aid the implementation of the intervention in their delivery settings (including resources and training)	Additional implementation strategies or actions developed to facilitate implementation in the different settings have been noted to influence success (15, 51, 52, 55). Strategies may include for example those for communication and engagement, stakeholder management, change management, resource and training or technological infrastructure (51, 52). Outlining the various strategies implemented along with their impact on the scale-up process as it will assist those wanting to develop similar strategies in the future.
6.5 Describe any modifications or adaptations required at the delivery setting/ organisation/ workforce level in order to scale-up the	Modifications to the delivery process and activities may be required in order to adapt to new contexts. Examples may include changing or redefining goals in one or more sites, changing funding support structures or making modifications in response to feedback from the settings

intervention. What were the reasons for modification? Were modifications applied universally across all sites/settings?	themselves. Adaptations may be necessary to facilitate the delivery of the intervention within the setting and recording these changes are important for understanding how the changes may or may not have contributed to ongoing sustainment of the intervention.
Section 7: The scale-up process<br>The purpose of this section is to document the scale-up process along with the scale-up workforce, resources available for managing and assisting with the scale-up process along with any evidence generated through this process.	
7.1 Describe the process undertaken to scale-up the intervention	The process undertaken for scale-up is complex and not frequently documented. While process models provide guidance on how to scale-up interventions (6, 7, 13, 14, 53, 54), in practice, the scale-up process is the different for every intervention (10). As socio-political contexts, target populations, availability of resources all vary across interventions and jurisdictions, so does the scale-up process. While the process steps as recommended in the scale- up models may be employed to improve the effectiveness of the delivery process, comprehensive reporting on the scale-up process will mean that lessons learnt from each experience can inform future attempts at scale-up and reduce duplication of mistakes. Future iterations of scale-up process models could also draw on these variations and incorporate more realistic scenarios and steps based on real-world experiences. Resources in the future can also be better utilised if known barriers are addressed early on due to adequate reporting of scale-up experiences that can be generalisable (34).
7.2 Describe the 'scale-up workforce' used to support the scale-up process. (i.e. where were human resources gathered, what were their roles and responsibilities?)	Detailed descriptions of the existence, structure and/or roles of the scale-up team, including how it was formed, the skillsets and roles of the personnel, as well as the available supporting resources or infrastructure can assist in informing future scale-up attempts and capacity for it to be replicated elsewhere (12, 77).
7.3 Describe any partnerships with other organisations to help support or manage the scale-up of the intervention	As noted in Section 6.3, partnerships/collaborations between multiple partners as a strategy to build capacity, resource or improve funding stability may have been formed to support, manage or even accelerate the scale-up process. Specifying the features of such partnerships if occurred within the scale-up workforce as noted previously, will assist in understanding these partnerships may occur in practice.

7.4 Describe the governance/ leadership and management structure directing the scale-up process	Effective governance and leadership has been noted to be an important facilitator to successful scale-up (8) therefore delineating and describing the specific structures and processes established to manage the scale-up process including documenting the various key actors and their roles and responsibilities will be helpful for those wanting to set up governance structures for future scale-up.
7.5 Describe any strategies that were used for scale-up (including communication strategies and advocacy)	In addition to the key steps taken to scale-up as documented in Section 7.1, additional implementation strategies or actions developed to facilitate the scale-up process has been noted to facilitate successful implementation (15, 51, 52, 55). These additional strategies such as those described in various implementation science frameworks may include strategies for communication and engagement, stakeholder management, change management, resource and training, technological infrastructure (51, 52). Outlining the various strategies developed and implemented along with their impact on the scale-up process as it will assist those wanting to develop similar strategies in the future.
7.6 Describe any barriers or facilitators in scaling up the intervention and strategies used to overcome barriers	A number of attempts have been made to document general facilitators and barriers to scale-up process (9, 19) and these may occur at any stage during the scale-up process. They may relate to the intervention and/or its components, the nature and structure of the scale-up settings or the delivery organisations, or even the scale-up workforce, the changing political context and priorities, key actors influencing the process or the scale-up process employed (9, 14, 19). Reporting the barriers and facilitators that contributed to the success or challenges to the intervention implementation at scale will help to improve implementation of interventions at scale more broadly.
7.7 Describe any strategies that have been developed to ensure the programs sustainability?	Strategic planning for the program’s long-term delivery has been identified as a core factor affecting a program’s capacity for sustainability (42). Details on any intentional or unintentional planning that was undertaken to guide the long-term direction, goals and strategies of the intervention in terms of resourcing, funding arrangements or stakeholder involvement, and where known, their impact on the intervention’s sustainability is useful for informing the development for future strategies to maximise intervention sustainability.

Section 8: Evidence of effectiveness<br>The purpose of this section is to describe any research and/or evaluation activities conducted during scale-up or post scale-up to determine the impact, outcome and/or effectiveness of the intervention. Research and/or evaluation into processes and implementation should also be documented.	
8.1 Describe the evidence generated during scale-up or post scale-up on the implementation, impact and/or outcome of the intervention at-scale.	Ongoing monitoring and evaluation of any intervention is critical to determining whether it is having its intended impact on the target population (74). This is just as critical if not more for an intervention that has been scaled up, where significant resources have been devoted to the process. The sustainability of interventions have been reported to be informed by such evidence generated through scale-up, with evidence of impact being a key consideration in ongoing funding decisions (8). As such, reporting on a) any of the formal monitoring and/or evaluation activities (such as formative, process, impact or outcome evaluations) that were conducted during or post scale-up and b) the impact of the outcomes of such monitoring and evaluation activities on the scale-up process, intervention and/or sustainability of the intervention will assist in understanding not only the impact of the intervention at scale but may provide reasons for its discontinuation or longer term sustainability.
8.2 Describe if any other interventions were de-implemented or modified as a result of the intervention that was scaled up.	In recent years, attention has been drawn to reducing the number of inappropriate or ineffective health interventions (78). The concept of de-implementation has been used to describe the process of removing interventions that are no longer deemed fit-for-purpose, required or superseded by other interventions or services (78). As with the implementation and scale-up of new interventions, there are a multitude of factors that impact on the de-implementation or modification of existing interventions. It is becoming increasingly important that this concept is acknowledged and conducted particularly if new interventions are being scaled up which then change the context for other interventions or services.
8.3 Describe the sustainability of the intervention post scale-up	Understanding the changes brought about by scale-up is key to facilitating long term sustainability (42, 79). The final stage of scale-up has often been described as institutionalisation, that is where a program or intervention has been successfully diffused into context and policy support and ongoing funding mechanisms have been established (74). Long term integration into an organisation’s functions and mandate may also be indicators of

	institutionalisation (74). Where this has occurred, it is important to document as features of the intervention's scale-up process may be examined to determine the key influencers of its success.

Case study S1: Two completed examples of scale-up using the Scale-up Reflection Guide

Example 1

Get Healthy Coaching Service: A free telephone-based service supporting the community to make sustained improvements in healthy eating, physical activity and maintaining a healthy weight.

The purpose of this example is to provide an illustration as to how the Scale-up Reflection Guide (SRG) could be used on real world interventions. As it is included as an Supplementary File to the study, it is not intended to be exhaustive, rather, it provides a brief overview of the types of information that can be reported within each of the sections.

**Section 1: SRG completion details**<br>The purpose of this section is to document when and how this SRG was completed.	
1.1 When the SRG was completed<br>1.2 Persons completing the SRG and affiliations<br>1.3 Main sources of information used for reporting this SRG	1.1 March – July 2020<br>1.2 University researcher (University of Sydney) with no personal involvement in the intervention (KL)<br>1.3 Publicly available documentation from peer-reviewed journals, various government documents, internet sources, qualitative interviews with program manager for the initial implementation phase of the intervention. Verification of the publicly available information on the current operations of the program was also provided by the current Manager of Operations of the GHS.
**Section 2: Information on the intervention**<br>The purpose of this section is to record the geographical location and scale-up approach. Information on the intervention components and characteristics is to be captured in Section 4.	
2.1 Location of the intervention (i.e. geographical location/s)<br>2.2 Level of scale-up achieved (i.e. city, state or national scale)<br>2.3 Time period of scale-up	2.1 New South Wales, Australia (primary); South Australia (previous) and Queensland (secondary/current)’ Australian Capital Territory (previous) and Tasmania (previous)<br>2.2 State-level, multiple states<br>2.3 Delivered at scale in February 2009 (commencing in NSW).<br>2.4 Vertical dissemination across the whole state population<br>2.5 Active

2.4 Type of scale-up approach taken<br>2.5 Current status of the intervention (active, no longer operational).	
**Section 3: Contextual and background information**<br>The purpose of this section is to document historical and contextual information underpinning the need to scale-up the intervention. This includes gathering information to:	
3.1 Describe the nature of the problem	Lifestyle related chronic diseases due to physical inactivity, sedentary behaviour and unhealthy eating are recognised as requiring population-scale health promotion programs through enabling infrastructure.
3.2 Describe the strategic and political context	In 2006, the Council of Australian Governments implemented a four-year $500 million national program - Australian Better Health Initiative (ABHI) to fund health promotion and prevention activities that specifically targeted lifestyle interventions and was to be implemented at the State/Territory level. The initiatives had to either scale-up existing programs or trial new innovative approaches. This national appetite for lifestyle interventions provided the opportunity for Get Healthy Service (GHS) and other pilot interventions of state jurisdictions to be considered for implementation or scale-up. The development of GHS required significant financial investment for at-scale delivery. A change in Federal Government in 2013, led funding of the ABHI to cease in 2014 budget and alternate sources of funding needed to be sourced within the State Health Budget for the program to continue.
3.3 Describe the strength of evidence of effectiveness that existed for the intervention	The evidence for mass-reach low-contact physical activity and nutrition behaviour interventions via telephone-based interventions had been systematically reviewed as effective in increasing physical activity, improving nutrition and reducing weight in the short to medium term (3-6 months). The development of GHS involved detailed research prior to implementation on the specific innovation and delivery aspects including the referral process, to the program content and delivery strategy. This involved stakeholder consultations (through focus groups with a large range of stakeholders) to work out the operational aspects of the intervention and compatibility (i.e. how it would be received by the population). Stakeholder consultations included target population and general community members, peak medical bodies, advocacy groups and service delivery personnel. The process of development was iterative with feedback from stakeholders reported to enhance the

	program development. Other existing telehealth services were also researched, including existing comparable services delivered by the State.	
3.4 Describe the decision-making process including documenting the key actors and their roles along with other factors that may have influenced this process	Funding support from the Federal Government was the key factor in the initial decision to implement the program. The stakeholder consultations helped to refine the intervention and identify delivery barriers. The consultation process also facilitated the 'buy-in' from all the key stakeholders. Key actors in the decision-making process included the health promotion program manager, high level executives and other stakeholders within the state Health Department, while key champions for the program included the Executive of the Health Promotion unit and other department heads. The key champions were strong advocates for the program and therefore supported its implementation. It was reported that Commonwealth funding would be used to finance the build of the initial infrastructure and once the intervention is able to demonstrate its effectiveness and reach, this would provide evidence for its continued funding. Director-General and Ministerial support was sought for the establishment of the service already advocating for population scale lifestyle interventions.	
Section 4: The intervention<br>The purpose of this section is to obtain key information about the intervention including its purpose, target audience and a description of the key elements. If the current scaled up intervention has changed from its original form or has been scaled up incrementally, it may be useful to divide this section into two or more columns as necessary to highlight the changes during scale-up.		
	**Current program (as at Sept 2020)**	**Original intervention in 2009**
4.1 Aims/objectives	The aim of the program was extended to include other lifestyle changes of alcohol reduction, and healthy weight gain during pregnancy	The GHS provided a free telephone based 6-month coaching service to support adults to make sustained improvements in healthy eating, physical activity, and achieving or maintaining a healthy weight.
4.2 Target population	The target population is similar to the original with the addition of the following subgroups:<br>• Pregnant or new mothers<br>• Alcohol dependent issues<br>• Aboriginal people	The Service was available for all adults residing in New South Wales, Australia (and subsequently other States/Territories that came on board at a later date) and who were at risk of developing chronic disease due to having one or more of the following risk factors: not meeting healthy eating guidelines; Inadequate physical

	And the modification of the target group to include all residents of participating States/Territories who are 16 years and over as of 2017.	activity or being overweight. This is a primary prevention intervention targeting whole of population aged 18 years and over.
4.3 Key intervention elements/components	GHS remains a free phone-based coaching service supporting adults make lifestyle changes regarding 5 specific modules: Healthy eating; Physical activity; Alcohol reduction; maintain a healthy weight and Healthy weight gain during pregnancy. GHI has two streams (a) tailored personal health coaching or (b) generic education information.<br><br>The health coaching component participants receive individually tailored phone coaching sessions (approximately 10 or 13) over a six-month period delivered by who qualified health professionals. A choice of 5 coaching programs is offered. In addition, Chinese speaking coaching (Cantonese or Mandarin) and a brief intervention (one off coaching call only) are now offered. The timings and frequency of the coaching calls are decided by between the participant and health coach, to enable them to be tailored to motivate and support participants to achieve their own healthy lifestyle goal. Coaching participants will also have access to free resources to record goals, progress and achievements. After the 6 months, participants can re-enrol in the Service or opt to continue coaching support via SMS through the Get Healthy Stay Healthy program. Participants can opt to receive information only. They will then receive free	In the beginning, GHS provided a free telephone based coaching service across two specific levels of service<br><br>• Information-only: Provides an evidence-based printed information package on healthy eating, physical activity, and achieving or maintaining a healthy weight.<br>• Six-month coaching program: Includes 10 individually tailored calls (over a 6-month period) provided by University qualified health coaches and are based on behaviour change/self-regulation principles designed to assist with goal setting, maintaining motivation, overcoming barriers and making sustainable lifestyle changes. Coaching calls are provided on a tapered schedule, with a higher intensity of calls occurring in the first twelve weeks of the program to promote initiation of behaviour change, and less frequent calls during the latter fourteen weeks to promote maintenance and prevent relapse. Participants are able to cease coaching at any time during the six-month program and are also able to re-enrol in the program after completing the six months.

	resources and a one-off phone coaching session. Information only participants can re-enrol in the Service as coaching participants at any point.	
4.4 Describe any modifications or adaptations required to the intervention components to enable scale-up	In some ways this intervention is continuing to be 'scaled up' and modified with new modules being added to the existing infrastructure/service. The new modules and services are added to include new population groups as well as new areas of need such as mental health, chronic or back pain. Adaptations to mode of delivery have also been occurring, for example, the inclusion of online resources to supplement the phone coaching service.	In 2012, there was a change made to reduce the requirement for medical clearance that made it easier to participate in the program thus reducing some of the barriers of the original program in 2009.<br><br>In 2012, the specific Aboriginal component of the program was also launched to specifically target the Aboriginal and Torres Strait Islander population.
Section 5: Intervention costs, funding and partnership arrangements<br>The purpose of this section is to document the costs, funding arrangements for the intervention being described. If over time, the funding provision or costs has changed it may be useful to divide this section into two or more columns as necessary to highlight the changes during scale-up.		
	**Current program (as at Sept 2020)**	**Original intervention in 2009**
5.1 Describe the cost(s) associated with the set up/ scale-up or delivery of the intervention.	In 2013-14, 1.8m and in 2017-18, 1.7m was provided for the Get Healthy Service in NSW.<br><br>Funding for service provision for the other States/Territories was provided by individual jurisdictions to NSW. The number of states dropped from five to two after the cessation of the NPAPH funding in 2014/15.	Approximately $1.8million for the first 18 months of operation.
5.2 Describe the source of funding, that is who provided the funding and	In NSW, the intervention is funded by the NSW Ministry of Health. Funding is provided as a grant from the Ministry of Health to HealthDirect (who is the	At the beginning, funding was obtained through the Commonwealth Better Health Initiative and later the National Partnership Agreement on Preventive Health.

any funding arrangements underpinning it (for example, co-funding, public private). Describe any changes to this funding source or arrangements over time.	third-party contractor engaged to manage the service). Within the NSW Ministry of Health, the Office of Preventive Health is the 'management group' that is responsible for the overall management of the contract and service. For services rendered in Queensland, the QLD government is provided an invoice, which is payable to NSW Ministry of Health.	Commonwealth funds ceased in 2014 and it is then fully funded by NSW Health.
**Section 6: The scale-up setting and delivery**<br>The purpose of this section is to document information on the setting in which the intervention was scaled up in as well as the delivery organisation and/or workforce employed. If over time, the setting has changed, it may be useful to divide this section into two or more columns as necessary to highlight the changes during scale-up.		
	**Current program (as at Sept 2020)**	**Original intervention in 2009**
6.1 Describe the setting the intervention was scaled up in.	The intervention is delivered to the NSW state population	The intervention was delivered at scale to the population of NSW. While self-referrals to the program was possible (and promoted through a public education campaign), local GPs and other health services were encouraged to refer suitable individuals to the program.
6.2 Describe the delivery organisation and/or workforce.	As at 2020, the service is managed by an external private company – outsourced by the state government. The coaching itself is delivered by university qualified health coaches. Individuals are able to refer themselves to the program or they may be referred by their health professional or local health service.	During the first 5 years from 2009 to 2013 the GHS was provided by a private company on behalf of the NSW Ministry of Health and from 2014 the service was provided by another private company.
6.3 Describe any partnerships that were formed to help	There is a three-way arrangement between a private organisation contracted by 'HealthDirect' to deliver	In the initial development phase, the GHS was managed out of a group within the Ministry of Health. It is likely

support or manage the delivery of the intervention	the service, and the NSW Ministry of Health who has a contract with 'HealthDirect' to manage the process. There are joint operational meetings to discuss implementation issues, and various adaptions and changes to implementation as and when required. Internally within the NSW Ministry of Health, the GHS is managed by the Office of Preventive Health (OPH) which an operational unit within the Ministry.	that the management of GHS was transferred to the Office of Preventive Health (OPH) when it was established in 2012.
6.4 Describe any implementation strategies that were used to aid the implementation of the intervention in their delivery settings (including resources and training)	One example of a training strategy were the regular education sessions of the telephone coaches on the different content areas for example, pain management, alcohol issues, physical activity and mental health to facilitate skill development and knowledge as well as to support research activity. This was often accompanied by minor changes to the IT infrastructure and system as well as updates and amendments to any supporting written materials.<br><br>Other strategies included regular communication strategies, which included low-cost social media strategies to connect with potential users and raise the profile of the service.	There were specific training programs (including evidence-based health coaching and motivational interviewing, cultural sensitivity training etc), policies and procedures, and quality control / assurance processes that were implemented and developed in collaboration with the provider, the MOH and the organisation contracted to conduct the evaluation of the service (University of Sydney).<br><br>The service was also supported by a public education campaign and a strategy to encourage referral through health practitioners and proactive marketing.
6.5 Describe any modifications or adaptations required at the delivery setting/ organisation/ workforce level in order to scale-up the intervention.	The operations centre of the telephone coaching service was initially based in Wollongong, NSW however, has since moved to be based in the Sydney CBD.	There were no significant changes to delivery setting and/or workforce were reported within the first five years of operation.
**Section 7: The scale-up process**<br>The purpose of this section is to document the scale-up process along with the scale-up workforce, resources available for managing and assisting with the scale-up process along with any evidence generated through this process.		

7.1 Describe the process undertaken to scale-up the intervention	The intervention was delivered 'at-scale' that is, was implemented state-wide following program development. It was deemed impossible given the type of intervention it was to conduct a pilot within a specific geographical area. (See Figure 1)
7.2 Describe the 'scale-up workforce' used to support the scale-up process.	The program scaled-up was managed by a central implementation team through the NSW Ministry of Health department.
7.3 Describe any partnerships with other organisations to help support or manage the scale-up of the intervention	The program scale-up was supported by evidence generation which was outsourced (but co-designed and co-managed).
7.4 Describe the governance/ leadership/ management structure of the scale-up process	A Clinical Advisory Group made up of NSW Ministry of Health clinicians, NGOs such as the Heart Foundation and Royal College of General Practitioners was established to advise on the development of coaching tools, contra-indications and medical clearance processes and clinical referral pathways.
7.5 Describe any strategies that were used for scale-up	• Having a centrally managed scale-up implementation team made it easier to have control over the processes as well as consistency.<br>• Referral targets placed into service level agreements with Local Health Districts to encourage referral to the service.<br>• A state-wide public education campaign and processes in relation to partnerships with key stakeholders such as GP NSW was implemented to support local efforts to drive traffic to the Service.<br>• Utilising the evaluation and monitoring processes / findings to facilitate continuous service improvements
7.6 Describe any barriers or facilitators in scaling up the intervention and strategies used to overcome barriers	**<u>Barriers</u>**:<br>• Mass-media campaigns were periodically implemented but only with modest budgets in the first five years. Subsequent campaigns focussed on increasing health professionals awareness of the service to improve referral rates.<br>• Lack of awareness among health professionals regarding the effectiveness of the service was a barrier as this meant that they were not actively referring appropriate individuals to the service. This was addressed through increasing marketing campaigns targeting health professionals.<br>**<u>Facilitators</u>**:

	• Extensive stakeholder consultation during the development of below the line marketing strategies including intervention – While this process assisted in refining the program and its components, it also engendered support and ownership in the intervention from all the stakeholders involved in the process.<br>• Comprehensive evaluation and monitoring – demonstrations of effectiveness were important
7.7 Describe any strategies that have been developed to facilitate the intervention's sustainability	Referrals to the Get Healthy Service from Local Health Districts were included in Performance Agreements will local Health Districts across the state. Referrals are monitored and targets set for referral to the service. The embedding of health service referrals as a routine part of clinical service delivery was important for sustainability and creating an ongoing demand for the service, this became particularly important as new modules were added to the GHS that required different target audiences to participate in the service (inclusion of secondary prevention level of program)<br><br>The robust evaluation and monitoring that were put in place early provide state level reporting against state health plans and demonstrate the program's effectiveness. Public circulation of the reports also provided public support and visibility.
**Section 8: Evidence of effectiveness**<br>The purpose of this section is to describe any research and/or evaluation activities conducted during scale-up or post scale-up to determine the impact, outcome and/or effectiveness of the intervention during or post scale-up. Research and/or evaluation into processes and implementation should also be documented. In this section, descriptions of longer-term outcomes resulting from the scale-up is also documented.	
8.1 Describe the evidence generated during scale-up or post scale-up on the implementation, impact and/or outcome of the scale-up and intervention.	A comprehensive evaluation framework was designed from the outset. There have been many evaluations conducted of the intervention as a whole and of different components of the intervention across the years since its implementation (Figure 1 below) These include: Process evaluation of advertising campaign; Evaluation framework; Characteristics of intervention participants; Evaluation of mass media communications ; Evaluation report 1; Evaluation report 2; Short term effectiveness; Maintenance of impact; Role of General Practice; Translational evaluation framework; Role of GHS with pregnant women; Aboriginal communities and Community mental health clients ; Chinese speaking community; Diabetes service; Tasmania Get Healthy Evaluation Report; Text messaging.
8.2 Describe if any other interventions were de-implemented or modified as	Not known

a result of the intervention that was scaled up.	
8.3 Describe the sustainability of the intervention post scale-up	The program continues to operate in 2020, embedded into the current state plans as a Premiers Priority.<br><br>The health service referrals as well as reporting to Local Health Districts has contributed to the services' sustainability. As a telephone service GHS has remained viable and critical during the COVID-19 Pandemic particularly during periods where restrictions to the home were in place.

References for this case study can be provided upon request to corresponding author.

Summary of Get Healthy Coaching Service scale-up timeline, milestones and research activities

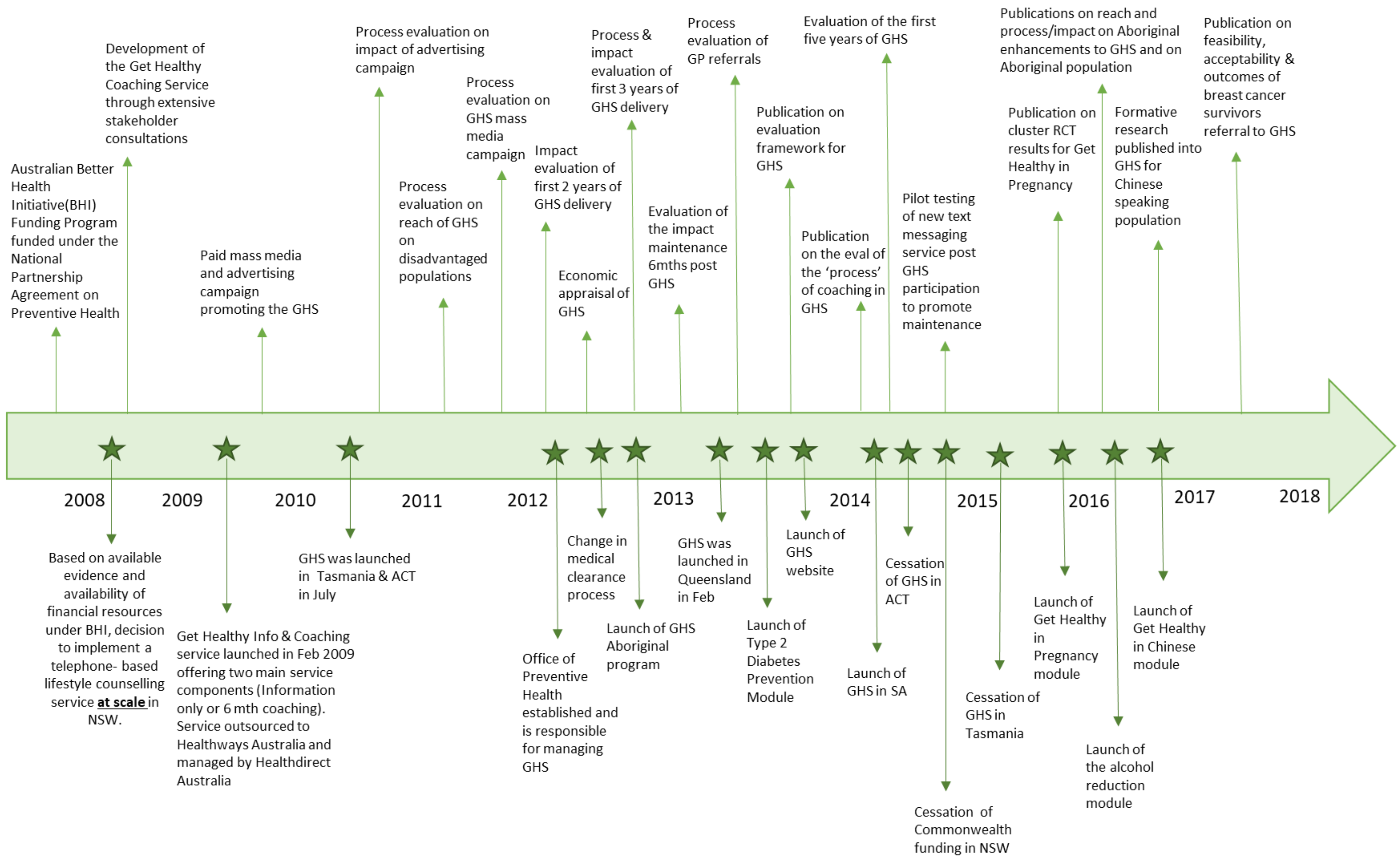

Example 2

Dutch Obesity Intervention in Teenagers (DOiT): A school-based intervention for children aged 12-14 years to prevent obesity by targeting energy balance-related behaviours.

The purpose of this example is to provide an illustration as to how the Scale-up Reflection Guide (SRG) could be used on real world interventions. As it is included as an Supplementary File to the study, it is not intended to be exhaustive, rather, it provides a brief overview of the types of information that can be reported within each of the sections.

**Section 1: SRG reporting details**<br>The purpose of this section is to document when and how this SRG was completed.	
1.1 When the SRG was completed.<br>1.2 Persons completing the SRG and affiliations.<br>1.3 Main sources of information used for reporting this SRG.	1.1 October 2019 – May 2020<br>1.2 University researcher (Amsterdam UMC) who conducted the implementation evaluation of Phase 2 (FvN)<br>1.3 Publicly available documentation from peer-reviewed journals, internet sources, qualitative interviews with initial developer (researcher), project manager at roll out organization, project manager at funder/roll out organisation.
**Section 2: Intervention geographical location and scale-up approach**<br>The purpose of this section is to record the geographical location and scale-up approach. Information on the intervention components and characteristics is to be captured in Section 4.	
1.1Location of the intervention (i.e. geographical location/s).<br>1.2Level of scale-up achieved (i.e. city, state or national scale).<br>1.3Time period of scale-up.	2.1 Netherlands<br>2.2 National scale<br>2.3 The intervention was developed and evaluated in an RCT effect evaluation (2003-2005), then delivered at scale during an implementation evaluation (2009-2013), and lastly rolled out by a commercial school-book publisher between 2013 to 2018. The roll out ceased in 2018.

1.4 Type of scale-up approach taken.<br>1.5 Current status of the intervention (active, no longer operational).	2.4 Vertical dissemination across the whole country. This was a research driven project, that then was transferred to practice.<br>2.5 The intervention is no longer operational (ended 2018).
**Section 3: Contextual and background information**<br>The purpose of this section is to document historical and contextual information underpinning the need to scale-up the intervention. If, over time the nature of the target problem and or intervention context has changed, it may be useful to divide this section into two or more columns as necessary to highlight the changes during scale-up.	
3.1 Describe the nature of the problem.	The program was developed to address the increasing number of children being overweight/obese. Pre-vocational students (aged 12-14 years) had higher levels of overweight/obesity and more unhealthy behaviours. Schools were regarded as a convenient and practical setting for implementing overweight prevention programs as they allowed access to almost all children and adolescents regardless of ethnic and socioeconomic background.
3.2 Describe the strategic and political context.	At the time of the initial intervention development (2001), there was only limited attention for obesity prevention in schools. During the implementation evaluation of Phase 2, DOiT aligned well with the Healthy School initiative, which was an initiative of the Dutch government in collaboration with various health organizations to promote healthy lifestyle in schools. This initiative was nationally implemented at many schools. This resulted in strong partnerships with the Dutch Heart Foundation, The Netherlands Nutrition Centre and Knowledge Centre for Sport & Physical Activity.
3.3 Describe the strength of evidence of effectiveness that existed for the intervention.	From 2003 to 2005, the programme was evaluated in a randomized controlled trial showing promising effects on adiposity measures (thinner skinfold thickness in girls and smaller waist circumference in boys) and EBRBs (a reduction of 250 ml in sugar-containing beverage consumption in both boys and girls, and a reduction in screen-viewing time of 25 min/day in boys). Using the RE-AIM framework, a process evaluation of DOiT was conducted. The majority of teachers regarded DOiT as suitable pre-vocational education material. Teachers reported that they planned to continue using DOiT and would recommend DOiT to other schools. This process evaluation also provided suggestions for further adaptation to DOiT, preparing the program for nationwide implementation.<br><br>The 2011-2013 implementation evaluation evaluated the impact of the DOiT program. From the total study sample, implementation of the adapted program did not lead to significant program effects on any of the

	adiposity measures (BMI, waist circumference or skinfolds) or target behaviours at 20-month follow-up (consumption of sugar-containing beverages (SCB), high-energy snacks/sweets and intake of breakfast, screen time (TV viewing and computer use), active transport to school and sport participation). However, subgroup analyses showed that the program resulted in significant beneficial effects on consumption of SCB in girls and breakfast consumption in boys. There was no evidence found for mediated intervention effects by the observed EBRBs on adiposity measures.<br><br>The accompanying process evaluation provided insight into the implementation 'black box' of the DOiT program. The percentage of accomplished implementation strategy activities ranged from only 9% (for 'closure meeting') up to 93% (for 'obtaining support within the school for adoption'). The percentage of lessons delivered decreased from 74 to 18% towards the end of the program. Fidelity to the teacher manual ranged from 26 to 85%. In general, teachers were satisfied with the DOiT lessons and teaching materials. Adolescents were moderately satisfied with the DOiT materials. Twenty-seven percent of the teachers at the 18 implementing schools reported that DOiT had become an embedded program in their school curriculum. Using an exploratory implementation index, a higher implementation index score was associated with program effectiveness; adolescents attending schools with a high implementation index score tended to have lower adiposity measures, while associations between implementation score and behavioural change were inconsistent.<br><br>No evaluation was conducted after this trial.
3.4 Describe the decision-making process including documenting the key actors and their roles along with other factors that may have influenced this process.	Funding support from the Dutch Heart Foundation was the key factor in the initial decision to develop the program, but also to support implementation beyond the first trial. The initial researchers played a key role in seeking funding for continued evaluation and implementation. As a result, new budget was available for research and implementation support. Due to the 'buy-in' from the Dutch Heart Foundation, The Netherlands Nutrition Centre and Knowledge Centre for Sport & Physical Activity the implementation during Phase 2 went well. Subsequently, funding ceased, and as part of consultations with different stakeholders, a 'schoolbook publisher', which is a private entity that supplies books to schools was selected to further continue the national roll out of DOiT, as it was supplementing their schoolbooks aimed at a healthy lifestyle for primary schools.
**Section 4: The intervention**	

The purpose of this section is to obtain key information about the intervention including its purpose, target audience and a description of the key elements.		
	**Intervention during dissemination (Phase 3 - 2013 - 2018)**	**Phase 1 intervention (2003-2006) & Phase 2 (2011-3013)**
4.1 Aims/objectives.	DOiT aimed to prevent obesity by targeting energy balance-related behaviours (EBRBs) among vocational students (12-14yrs).<br><br>The intervention targeted the following EBRBs: sport participation, active transport, computer/screen time, consumption of snacks, sugar-containing beverages and eating a healthy breakfast.	DOiT aimed to prevent obesity by targeting energy balance-related behaviours (EBRBs) among vocational students (12-14yrs).<br><br>The intervention targeted the following EBRBs: sport participation, active transport, computer/screen time, consumption of snacks, and sugar-containing beverages.<br><br>Initially eating a healthy breakfast was not included into the program, but by revisiting the development process in 2009 (Phase 2) it became clear that this behaviour needed to be included as well.
4.2 Target population.	Although the intervention was specifically developed to target pre-vocational education students, many schools expressed the need to not only deliver the program to their pre-vocational students, but also to the higher levels of secondary school that were also present in the same school.<br><br>The schoolbook publisher correspondingly offered the program to all secondary schools as well, not just pre-vocational schools.<br><br>Reach Phase 3: Unknown.	The program was initially developed for 12-14 years old students attending the first and second year of pre-vocational education.<br><br>Reach Phase 1: After being further informed about the requirements of participation in the study, 18 schools were willing to participate.<br><br>Reach Phase 2: Recruitment of schools took place from January 2011 to December 2011 (114). In total, n= 628 contacts (i.e. teachers and professionals such as intermediaries at municipalities, municipal health services or sport organizations) were approached by

		email and/or mail. Because of the nature of the programme rollout, the denominator (i.e. how many schools were approached in total) was unknown. Overall, n=66 schools ordered the DOiT materials within the first year. Twenty-six schools (39%) ordered the programme themselves and 40 schools (61%) received the programme via local supporting organizations (e.g. intermediaries ordered materials for the school).<br><br>In the period 2011-2013 89 schools ordered the program resulting in reaching >11,000 students.
4.3 Key intervention elements/components.	The intervention consisted of 16 lessons divided over two school years; 12 theory lessons delivered by teachers during Biology, Health care or Mentor lessons, and 4 PE lessons delivered by PE teachers.	The intervention was systematically developed according to the Intervention Mapping Protocol, using the input of both theory and end-user/stakeholders.<br><br>The initial 2002 version of DOiT included different theoretical methods and practical strategies translated into the DOiT materials to promote healthy EBRBs in 11 lessons incorporated in biology and physical education (PE) lessons during one school year. The environmental component included advice to the school staff for changes in school canteens and financial support to provide additional physical activity options in the school setting.<br><br>The adapted 2009 program consisted of 12 fixed theory lessons and four physical education lessons (i.e. 16 lessons equally divided over two school years), the environmental was adapted and a parental component

		was added. The DOiT materials included a 'schoolbook' accompanied by separate worksheets, a student toolkit (pedometer, food/exercise diary and online computer-tailored advice) and a parental information booklet.<br><br>Schools could substitute the regular Biology, Health Education and physical education (PE) lessons dealing with healthy nutrition and physical activity behaviour by the DOiT lessons.<br><br>To provide flexibility, additional materials and suggestions were provided in the teacher manual and online resources to allow for tailoring to a specific school context or facilities.
4.4 Describe any modifications or adaptations required to the intervention components to enable scale-up.	The schoolbook publisher made minor modifications to the layout and format of the intervention resources along with some minor changes to the text. They also decided not to provide schools with the implementation plan and support, which was part of the initial stages of implementation. Similarly, in the first year, they had employed personnel whose was tasked with promoting the sale of the program to schools, however due to other duties, the task of promoting the program to schools ceased.	During the 2009 development process a 7-step implementation strategy for teachers was developed. The adapted website provided general information about the DOiT program, such as contact information, news items, a list of DOiT schools and a promotional video. The website consisted of four domains targeting teachers, health promotion professionals, parents and adolescents. This website provided a toolkit for implementation of DOiT containing materials for each implementation step to facilitate the implementation process, such as factsheet, brochure, exemplary teaching materials, prefabricated presentation, plan for implementation, instructional video and evaluation guideline.

		Furthermore a 'DOiT support office' was installed at the University, to support implementation schools and actively recruit schools by sending a DOiT introductory package existing of an information letter with a factsheet, brochure and exemplary teaching materials. Additionally, different promotion activities, such as news items on different relevant websites and in digital mailings, presentations at national conferences and local meetings of different stakeholders, were executed in order to reach potential users. Health promotion professionals were also actively contacted for their participation in recruitment of schools.<br><br>In addition, DOiT was submitted to the national intervention database. This database provides policy makers and health promotion professionals with information on the quality and effectiveness of available health promotion interventions in order to promote use of evidence-based interventions. Since most stakeholders emphasized that registration of DOiT in the national database would be helpful to distinguish DOiT from other "similar" programmes, DOiT was submitted for the Dutch registration database. DOiT was labelled as theoretically sound.
Section 5: Intervention costs, funding and partnership arrangements<br>The purpose of this section is to document the costs, funding arrangements for the intervention being described.		
	**Intervention during dissemination (Phase 3 - 2013 - 2018)**	**Phase 1 intervention (2003-2006) & Phase 2 (2011-3013)**

5.1 Describe the cost(s) associated with the set up/ scale-up or delivery of the intervention.	Schools were able to purchase the interventions themselves with the schoolbook publisher. Support costs were unknown.	During Phase 1, implementation costs were fully covered by the research funding of the Dutch Heart Foundation (€270k research budget).<br>During Phase 2, school could buy the intervention for 7 euro per student for the two-year programme. Schools who participated in the evaluation received the materials for free for three classes, additional materials or other schools could buy the materials at the DOiT office, hosted at the university.
5.2 Describe the source of funding and any funding arrangements underpinning it (e.g., co-funding, public private). Describe any changes to this funding source or arrangements over time.	Unknown.	During Phase 2 funding of the Dutch Heart Foundation (€180k), The Netherlands Nutrition Centre (in-kind) and Knowledge Centre for Sport & Physical Activity (€40k) covered costs for development, evaluation, dissemination, and implementation support.<br>In addition, €200k was funded by the SNS REAAL Fonds (Netherlands) to evaluate the nationwide implementation of the DOiT program.
Section 6: The scale-up setting and delivery<br>The purpose of this section is to document information on the setting in which the intervention was scaled up in as well as the delivery organisation and/or workforce employed. If over time, the setting has changed, it may be useful to divide this section into two or more columns as necessary to highlight the changes during scale-up.		
	**Intervention during dissemination (Phase 3 - 2013 - 2018)**	**Phase 1 intervention (2003-2006) & Phase 2 (2011-3013)**
6.1 Describe the setting the intervention was scaled up in.	All secondary schools in the Netherlands	Pre-vocational education schools in the Netherlands.

6.2 Describe the delivery organisation and/or workforce.	Biology, health care or mentor teachers, and PE teachers.	Biology, health care or mentor teachers, and PE teachers.
6.3 Describe any partnerships that were formed to help support or manage the delivery of the intervention.	Unknown.	During the scale up research, the researchers collaborated with municipal health services.
6.4 Describe any implementation strategies that were used to aid the implementation of the intervention in their delivery settings.	Schools received a short manual. No additional online resources were provided. No training was provided.	During Phase 1, schools received a manual for implementation and brief face-to-face training.<br><br>During the Phase 2, an extensive teacher manual was developed in order to guide teachers through the complexity of implementing the different program components. Accompanied with the teacher manual, a login code for the DOiT website was provided. Once logged-in on the DOiT website, teachers had access to the short instructional video, worksheets and additional lessons.<br><br>For each lesson more interactive materials were provided as a replacement for parts of the DOiT lessons, anticipating on variability in schools.<br><br>Logging-in enabled teachers to access the online teacher area, where teachers can watch the instructional video and download worksheets as well as additional materials.<br><br>Other strategies included regular communication strategies by the DOiT office, and PR activities to connect with potential users.

<table>
<tr><td>6.5 Describe any modifications or adaptations required at the delivery setting/ organisation/ workforce level in order to scale-up the intervention.</td><td>During Phase 3, the schoolbook publisher kept the program and delivery methods mostly the same.</td><td>After Phase 2, a small grant (€3k) was obtained to explore scale up possibilities beyond the research budget. Interviews with different stakeholders resulted in the selection of the schoolbook publisher to roll out the program, based on their view on the program and potential for roll out, as well as compatibility with their other school books. A contract was signed for a few years.</td></tr>
<tr><td colspan="3">Section 7: The scale-up process
The purpose of this section is to document the scale-up process along with the scale-up workforce, resources available for managing and assisting with the scale-up process along with any evidence generated through this process.</td></tr>
<tr><td>7.1 Describe the process undertaken to scale-up the intervention.</td><td colspan="2">Phase 2: In order to reach all Dutch pre-vocational schools, a recruitment strategy was developed. Following this strategy, the ‘DOiT support office’ actively recruited schools by sending a DOiT introductory package existing of an information letter with a factsheet, brochure and exemplary teaching materials. Additionally, different promotion activities, such as news items on different relevant websites and in digital mailings, presentations at national conferences and local meetings of different stakeholders, were executed in order to reach potential users. Health promotion professionals were also actively contacted for their participation in recruitment of schools.

Phase 3: The schoolbook publisher had a recruitment and promotion person (only few hours per week) to reach out to schools directly, but also to municipal health services. The municipal health service was described as one of the biggest facilitators for the dissemination of DOiT as they reached out to schools to deliver the intervention. As the municipal health service had close contact with schools, they were able to distribute the interventions to the schools which facilitated the dissemination.</td></tr>
<tr><td>7.2 Describe the ‘scale-up workforce’ used to support the scale-up process.</td><td colspan="2">Phase 2: The DOiT office actively supported schools, in addition to all support materials on the DOiT website.

Phase 3: The employee at the schoolbook publisher only provided minimal support to schools, their main focus was on recruitment.</td></tr>
</table>

7.3 Describe any partnerships with other organisations to help support or manage the scale-up of the intervention.	Phase 2: Strong partnerships with the Dutch Heart Foundation, The Netherlands Nutrition Centre and Knowledge Centre for Sport & Physical Activity helped to support and manage the scale up of DOiT. They had devoted personnel that closely collaborated with the DOiT office and also used their communication channels to reach new users.<br><br>Phase 3: Unknown.
7.4 Describe the governance/ leadership and management structure directing the scale-up process.	During Phase 2 there was a Steering Committee consisting of representatives of the Amsterdam UMC, the Dutch Heart Foundation, The Netherlands Nutrition Centre and Knowledge Centre for Sport & Physical Activity. They supervised the research project and advised on actions and strategies for scaling up.
7.5 Describe any strategies that were used for scale-up (including communication strategies and advocacy).	Phase 2: See Section 6.4.<br><br>Phase 3: Only limited scale-up strategies were in place (support staff and information on website).
7.6 Describe any barriers or facilitators in scaling up the intervention and strategies used to overcome barriers.	Phase 2: During implementation evaluation various important implementation barriers were identified (e.g. lack of planning, other urgent unforeseen priorities, no plan to cope with teacher turnover and high teacher workload) as well as facilitating factors (e.g. involvement of DOiT coordinator and support from the DOiT office, sufficient communication and collaboration between teachers, strong teacher motivation and flexibility of the programme).<br><br>Phase 3: After initial research funding, DOiT developed their scale-up revenue model around the sale of the program to schools by a schoolbook publisher. Interviewees described that this revenue was needed for appointing supportive staff as well as ongoing development to prevent that the intervention would become too outdated. A strategy in this revenue model consisted of combining the textbook and workbook. However, in practice this model did not work due to low prices as well as lack of urgency by schools to buy the program. Lack of urgency was partly caused by schools copying the books and not buying them annually. Therefore, lack of generated revenue for the schoolbook publisher was mentioned as a barrier in enhancing and continuation of the intervention scale up.

	Another barrier identified was that before DOiT became property of the schoolbook publisher, the previous owners (i.e. researchers) had already provided a large number of schools with free or reduced price materials which resulted in not many schools wanting to buy the new materials from the publisher anymore. The potential market for the intervention was described as significantly reduced, consequently causing an insufficient number of schools buying the materials from the publisher and hindering scale up.
7.7 Describe any strategies that have been developed to ensure the intervention's sustainability.	Phase 2: DOiT was submitted to the Dutch registration database that grants programmes to one of four possible levels of recognition depending on a rating of the evidence base for the programme (i.e. theoretically sound, probable effectiveness, established effectiveness, and established cost effectiveness). This database provides policy makers and health promotion professionals with information on the quality and effectiveness of available health promotion interventions in order to promote use of evidence-based interventions. Since most stakeholders emphasized that registration of DOiT in the national database would be helpful to distinguish DOiT from other "similar" programmes, we submitted DOiT for the Dutch registration database. DOiT was labelled as theoretically sound.<br><br>A facilitator identified was correspondence of intervention with other ongoing health policies. Healthy School was an initiative of the Dutch government in collaboration with various health organizations to promote healthy lifestyle on schools. Participants described that once schools had already adopted Healthy School policies into their school, they were more likely to adopt DOiT as well, since the intervention aligned well with this policy.
**Section 8: Evidence of effectiveness and long-term outcomes**<br>The purpose of this section is to describe any research and/or evaluation activities conducted during scale-up or post scale-up to determine the impact, outcome and/or effectiveness of the intervention. Research and/or evaluation into processes and implementation should also be documented. In this section, descriptions of longer-term outcomes resulting from the scale-up should also documented.	
8.1 Describe the evidence generated during scale-up or post scale-up on the implementation, impact and/or outcome of the intervention at-scale.	Evaluation results during Phases 1 and 2 are described in 3.3<br><br>During Phase 3 no evaluations were conducted.
8.2 Describe if any other interventions were de-	Not known.

implemented or modified as a result of the intervention that was scaled up.	
8.3 Describe the sustainability of the intervention post scale-up	The scale up of the intervention ceased in 2018.

References for this case study can be provided upon request to corresponding author.

Summary of Dutch Obesity Intervention in Teenagers (DOiT) Phases

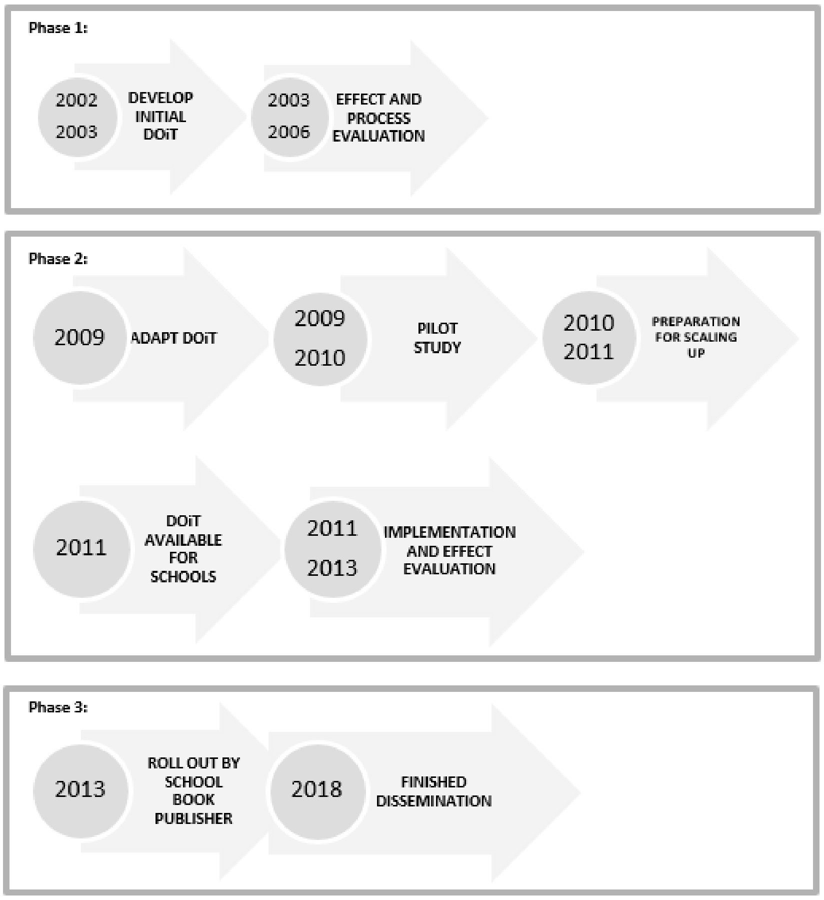

Supplement: Supplementary file 1 [file ijerph-20-06014-s001.zip › ijerph-2313631-supplementary.pdf]
